# Climate impacts and Arctic precursors of changing storm track activity in the Atlantic-Eurasian region

**DOI:** 10.1038/s41598-018-35900-8

**Published:** 2018-12-12

**Authors:** Pawel Schlichtholz

**Affiliations:** Institute of Oceanology, Polish Academy of Sciences, Powstancow Warszawy 55, 81-712 Sopot, Poland

## Abstract

Midlatitude storm tracks are preferred regions of intense activity of synoptic eddies shaping the day-to-day weather and several aspects of surface climate. Here statistical analyses of observationally-based atmospheric data and observed Arctic sea ice concentration (SIC) in the period 1979–2017 are used to identify linkages of a dominant mode of interannual variability in wintertime upper-tropospheric storm track activity over Eurasia (STA_EA_ mode) to the concurrent surface climate anomalies and pre-winter Arctic SIC variations. This mode explains an exceptionally large fraction (about 70% of the variance) of the North Atlantic Oscillation (NAO) and of a dominant mode of Eurasian surface air temperature variations. As more than 50% of the variance of the STA_EA_ mode and NAO is found to be accounted for by October SIC anomalies in the Barents/Kara Sea, it is concluded that wintertime Eurasian climate variability is to some extent predictable and that this predictability might have increased after an acceleration of the sea ice cover decline in the mid 2000s. These conclusions are supported by results from leave-1-yr-out cross-validated forecast experiments.

## Introduction

**Figure 1:**
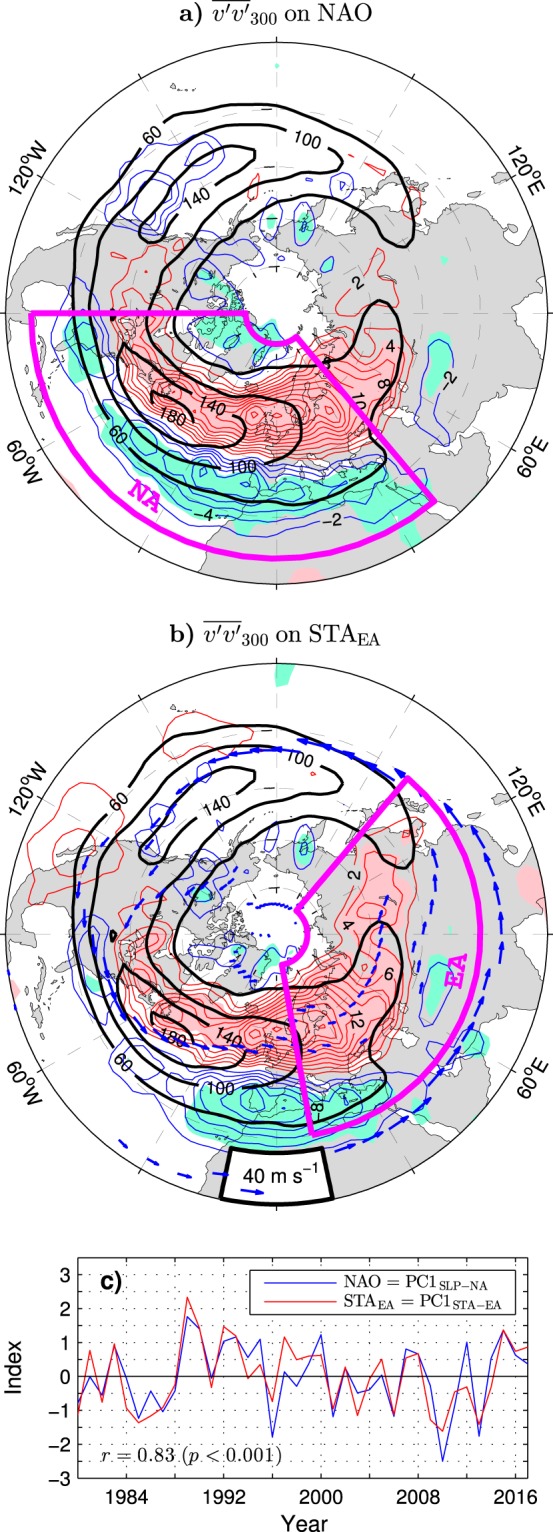
Relation between the wintertime (DJFM mean) NAO index and concurrent variations of storm track activity in the ESO period. (**a** and **b**) (Thin contours and color shading), Detrended anomalies of ${\overline{v^{\prime} v^{\prime} }}_{300}$ regressed onto the NAO and STA_EA_ indices, respectively. Red (blue) contours represent positive (negative) anomalies. The contour interval (CI) is 2 m^2^ s^−2^ per unit index. Pink (aquamarine) shading denote positive (negative) anomalies statistically significant at the 95% confidence level. Thick black contours show the wintertime climatology of ${\overline{v^{\prime} v^{\prime} }}_{300}$ (in m^2^ s^−2^). In (**b**) blue arrows depict the wintertime climatology of jet streams. (**c**) Comparison of the NAO (blue curve) and STA_EA_ (red curve) indices (years correspond to the January). The NAO index is the PC time series of the first leading mode of SLP variability in the North Atlantic region (magenta box in (**a**)). The STA_EA_ index is the PC of the first leading mode of ${\overline{v^{\prime} v^{\prime} }}_{300}$ variability over Eurasia (magenta box in (**b**)). Both indices are based on detrended data. The maps were generated by MathWorks MATLAB R2014a with M_Map (http://www.eoas.ubc.ca/rich/map.html).

The extratropical weather and climate at the earth’s surface and throughout the troposphere (about 10-km-thick, lowermost layer of the atmosphere) depend profoundly on formation, propagation and decay of midlatitude, synoptic-scale, short-lived storms (cyclones)^1–3^. Most intense storms develop in the cool season, typically via baroclinic instability in the lower troposphere^4^. In the Northern Hemisphere (NH; see Supplementary Table S1 for a summary of acronyms used in this paper), main “storm production centers” are located just off the east coasts of North America and Eurasia. Storm activity is generally transferred from these centers upward and eastward towards the continents^5,6^. The storms finally decay by releasing their energy to jet streams, that is, high-speed cores of predominantly westerly mean winds in the upper troposphere and through mechanical dissipation at the surface^2^. On the daily-to-weekly timescale, the energy of individual storms fuels extreme weather events such as damaging winds^7^ and floods^8^. On longer timescales, poleward heat, moisture and momentum transport by transient eddies (cyclones and anticyclones) stabilises the Equator-to-pole energy gradient^9^ and helps to maintain the general atmospheric circulation against dissipation^10^. Preferred regions of high synoptic eddy activity, commonly referred to as “storm tracks”, are easily identified as regions with large variances and covariances of atmospheric quantities (filtered to retain only the weather signal)^11,12^. The anatomy of upper-tropospheric storm tracks in the NH extratropics, with peaks over midlatitude oceans marking the Pacific and Atlantic storm tracks, is depicted by black contours in Fig. 1a,b showing wintertime climatology of the storm track activity (STA) defined as the variance of the 2–6 day bandpass-filtered meridional wind at 300 hPa (${\overline{v^{\prime} v^{\prime} }}_{300}$, see Methods). The corresponding location of jet streams is indicated by blue arrows in Fig. 1b (see Methods for the algorithm of their detection).

Owing to a symbiotic coexistence of storm tracks, jet streams and planetary waves^13–15^, changes in frequency or intensity of synoptic eddies and subtle shifts of their preferred paths can generate considerable flow anomalies impacting the climate both locally and on the planetary scale. Specifically, major low-frequency teleconnection patterns, such as the most prominent regional mode of wintertime variability of the tropospheric circulation in the NH extratropics, known as the North Atlantic Oscillation (NAO)^16^, have been shown to be related to storm-track changes^17–19^. Inarguably, in the era of satellite observations (ESO period), the Atlantic storm track has been migrating north and south in tune with the NAO polarity, as demonstrated by NAO-related wintertime anomalies of ${\overline{v^{\prime} v^{\prime} }}_{300}$ in Fig. 1a (thin contours and color shading).

Mechanisms shaping variability of the midlatitude storm tracks are complex. They include not only feedbacks between tropospheric processes on multiple spatial and temporal scales but also interactions with the underlying surface^20^ and the overlying stratosphere^21^, as well as interactions with the tropical^22,23^ and polar^24^ atmosphere. In particular, a tug of war between opposing influences on air temperature gradients makes projections of future location and intensity of storm tracks instigated by anthropogenic climate change very uncertain^3^. Identification and understanding of predictability of the midlatitude storm tracks arising from natural climate variations on short (seasonal to decadal) timescales are also challenging. Ensemble hindcasts from a high-resolution climate model show that seasonal mean storm tracks can be skillfully predicted a few months ahead and that this predictability is related, in the first place, to the tropical phenomenon of El-Niño-Southern Oscillation^25^. In boreal winter, the most predictable signal is found over North America while the predictable signal over Eurasia appears to be small. This does not, however, mean that seasonal predictability of storm tracks over Eurasia is negligible since the analysis in ref.^25^ is limited to two storm track components that maximise the predictability globally. Moreover, these components are derived from a surface storm track statistic, which may not be an optimal measure of storm track variations over Eurasia. Ensemble hindcasts from the same climate model demonstrate a significant skill in prediction of seasonal mean surface climate variables, such as land temperature and precipitation, in both North America and Eurasia^26^.

Numerous studies have shown more or less significant relations of wintertime climate variability over Eurasia to preceding summer and autumn Arctic sea ice cover^27–31^ and many of them recognised the importance of storm tracks in shaping these relations (see refs^24,32–35^ for reviews). However, none of them treated explicitly predictability of storm tracks provided by Arctic sea ice cover variations. On the other hand, there is increasingly more evidence that considerable seasonal predictability of the winter NAO exists^36^ owing to its response to slow changes in the stratosphere^21,37^ and lower boundary conditions, including sea surface temperature in the North Atlantic^38^, Eurasian snow cover^39,40^ and Arctic sea ice cover^29,30^. A recent study demonstrated a skillful seasonal empirical prediction of a winter mean NAO index from a combination of these predictors^41^ and found that Arctic sea ice cover anomalies may be the most efficient source of the NAO predictability. Moreover, some aspects of non-NAO-controlled Eurasian climate variability in winter, including storm track variations, are significantly related to earlier ocean heat anomalies in the pathway of warm Atlantic water to the cold Arctic Ocean^42^. Therefore, wintertime storm track statistics in the Atlantic-Eurasian region should be to some extent predictable.

The present study, based on statistical analyses, investigates interannual variability of the winter (December-to-March; DJFM) mean storm tracks in the Atlantic-Eurasian region and its link to concurrent variations of atmospheric circulation and earlier anomalies of Arctic sea ice cover during the ESO period (1979–2017). A significant link of the NAO to a leading mode of STA variability in the North Atlantic region (NA box in Fig. 1a) is found. This link is then optimised by inspecting its sensitivity to the choice of the geographical region for computation of the leading STA mode. A particularly strong relation to the NAO is detected for the mode computed over Eurasia (EA box in Fig. 1b). Upper-tropospheric circulation and surface air temperature (SAT) anomalies associated with the time series of this mode, hereafter referred to as the STA_EA_ index, suggest that downstream NAO teleconnections to SAT variability in northern Asia^43–45^ reflect interactions between the storm tracks and a high-latitude quasi-stationary planetary wave. These interactions should be steered to some extent by external forcings, as indicated by a significant linkage of the STA_EA_ mode and the related surface climate variability to pre-winter sea ice concentration (SIC) anomalies in the Barents/Kara Sea region. Based on forecast experiments, it is demonstrated that this linkage provides a useful amount of predictability of wintertime climate variations in Eurasia and that this predictability might have recently strengthened in association with accelerated Arctic sea ice decline.

## Wintertime Storm Track Variability

### Leading mode in the North Atlantic region

**Figure 2:**
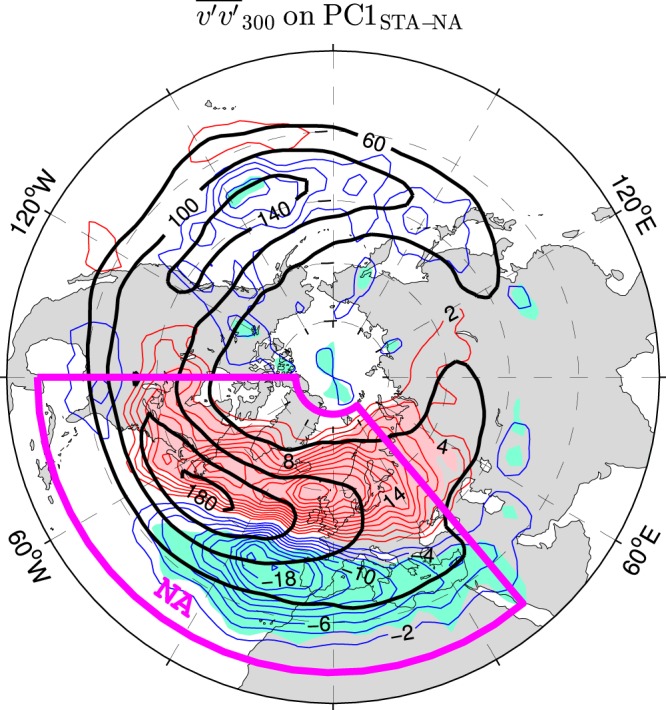
Leading mode of wintertime storm track activity variations in the North Atlantic region during the ESO period. Thin contours and color shading show anomalies of ${\overline{v^{\prime} v^{\prime} }}_{300}$ regressed onto the PC time series (PC1_STA–NA_) of the first mode of ${\overline{v^{\prime} v^{\prime} }}_{300}$ variability in the North Atlantic region (marked by a magenta box). PC1_STA–NA_ and the regressed field are based on raw (nondetrended) data. The CI is 2 m^2^ s^−2^ per unit PC1_STA–NA_. The thin contour and shading colors are explained in the caption to Fig. 1. Thick black contours show the wintertime climatology of ${\overline{v^{\prime} v^{\prime} }}_{300}$ (in m^2^ s^−2^). The map was generated by MathWorks MATLAB R2014a with M_Map (http://www.eoas.ubc.ca/rich/map.html).

Leading modes of wintertime atmospheric variability in the ESO period are extracted from NCEP/NCAR reanalysis data using the empirical orthogonal function (EOF) technique (see Methods). The anomaly pattern of ${\overline{v^{\prime} v^{\prime} }}_{300}$ regressed onto the principal component (PC) time series of the first mode of STA variability in the North Atlantic region derived from the raw (nondetrended) data is shown in Fig. 2 (thin contours and color shading). This pattern resembles the anomaly pattern of the second (“latitudinally shifting”) mode of month-to-month STA variability in the North Atlantic region based on data from the 40-yr European Centre for Medium-Range Weather Forecasts (ECMWF) Re-Analysis (ERA-40) in the period 1958–2001^46^. Both patterns exhibit longitudinally elongated dipoles that straddle the climatological Atlantic storm track, with main centers of action located downstream (eastward) of the climatological maximum (see the black contours in Fig. 2 for the wintertime STA climatology in the ESO period).

The first mode of the wintertime STA variability in the North Atlantic region during the ESO period is not statistically reliable irrespective of whether or not the time series are detrended prior to the EOF decomposition. This is indicated by the metric *s* expressing North’s “rule of thumb”^47^ (see Methods for computational details). This metric is included in Supplementary Table S2 summarising statistics of selected EOFs based on raw data and in Supplementary Table S3 summarising statistics of selected EOFs based on detrended data. For the first mode of the STA variability in the North Atlantic region, *s* largely exceeds one, meaning that this mode is not separated significantly from the second mode. A physical relevance of this mode is, however, suggested by a strong covariability of its time series (PC1_STA–NA_) with the NAO index defined as the PC time series of the leading mode of the wintertime sea level pressure (SLP) variability computed for the same (North Atlantic) region and the same (ESO) period (PC1_SLP–NA_). This covariability is strong for the leading STA and SLP modes based on the detrended data (*r* = 0.75; see Supplementary Table S3) as well as raw data (*r* = 0.74; see Supplementary Table S2). It is also robust with regard to the definition of the NAO index. Indeed, PC1_SLP–NA_ captures very well (*r* > 0.95) the variability represented by other commonly used NAO indices based on SLP anomalies (see Methods for their definition and Supplementary Fig. S1 for comparison of the detrended time series).

The relation between PC1_SLP–NA_ and PC1_STA–NA_ is tighter than previously reported relations between NAO indicators and indices of STA variability in the North Atlantic region calculated from reanalysis data for either longer^46^ or shorter^48^ periods. More important, there are qualitative differences resulting from either different periods considered or different timescales represented by the data used in the EOF analysis. For instance, while the wintertime NAO index in the ESO period covaries significantly with the “shifting” mode of the corresponding STA variability, the month-to-month NAO index in the 1958–2001 period covaries significantly with a “pulsating” mode of the corresponding STA variability based on the ERA-40 data^46^.

### Leading mode in the Eurasian sector

Even though the first mode of the wintertime STA variability in the North Atlantic region during the ESO period is strongly linked to the NAO, this mode is not optimal for analysing the STA-NAO relationship. A stronger relation to the NAO is found for the leading mode of the wintertime STA variability over extratropical Eurasia (EA box in Fig. 1b). The PC time series of this mode (PC1_STA–EA_ or STA_EA_ index) computed from detrended data of ${\overline{v^{\prime} v^{\prime} }}_{300}$ correlates with the NAO index at 0.83 (see Fig. 1c for comparison of the time series). Given such a close relationship, the anomaly patterns of ${\overline{v^{\prime} v^{\prime} }}_{300}$ regressed onto the NAO and STA_EA_ indices strongly resemble one the other (compare thin contours in Fig. 1a,b). During the positive phase of these indices, the Atlantic storm track migrates north and extends farther east. Maximum STA anomalies in the northern and southern lobes of the anomalous dipole straddling the climatological storm track are found around the Baltic Sea area and in the Mediterranean Sea region, respectively.

**Figure 3:**
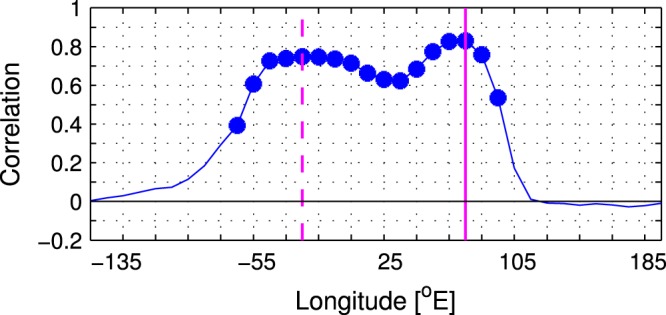
Longitudinal dependence of the correlation coefficient between the principal component time series (PC1) of the leading regional mode of wintertime storm track activity variations and the concurrent NAO index in the ESO period. The PC1’s of STA variability are computed from detrended anomalies of ${\overline{v^{\prime} v^{\prime} }}_{300}$ over a moving box in the latitudinal band 30°–80°N that is 130° wide in longitude. The box is moved circumglobally with a step of 10° of longitude. The abscissa indicates the central longitude of the box. The full and dashed magenta vertical lines indicate the central longitude of the Eurasian sector (10°–140°E) and the North Atlantic sector (90°W–40°E), respectively. The filled circles denote correlations statistically significant at the 95% confidence level.

The relation of the NAO index to the STA_EA_ index is tighter than its relation to the PC1 of the wintertime STA variability calculated for any other sector having the same area as the Eurasian (EA) box in Fig. 1b. This is demonstrated in Fig. 3 displaying the running correlation of the NAO index with the full “circumglobal” set of STA PC1’s computed in the latitudinal band from 30° to 80°N for 130°-wide boxes, each shifted by 10° of longitude with respect to the adjacent one. The correlation exhibits an absolute maximum (*r* = 0.83) just at the central longitude of the Eurasian box in Fig. 1b (75°E) and a secondary maximum (*r* = 0.75) at the central longitude of the North Atlantic box in Fig. 1a (25°W). The STA_EA_ index is selected for the following analysis since the leading mode of STA variability in the Eurasian sector is not only tightly linked to the NAO but also independent from the corresponding second mode (*s* < 1; see Supplementary Table S3).

## Relation to Concurrent Climate Variations

### Circulation anomalies

**Figure 4:**
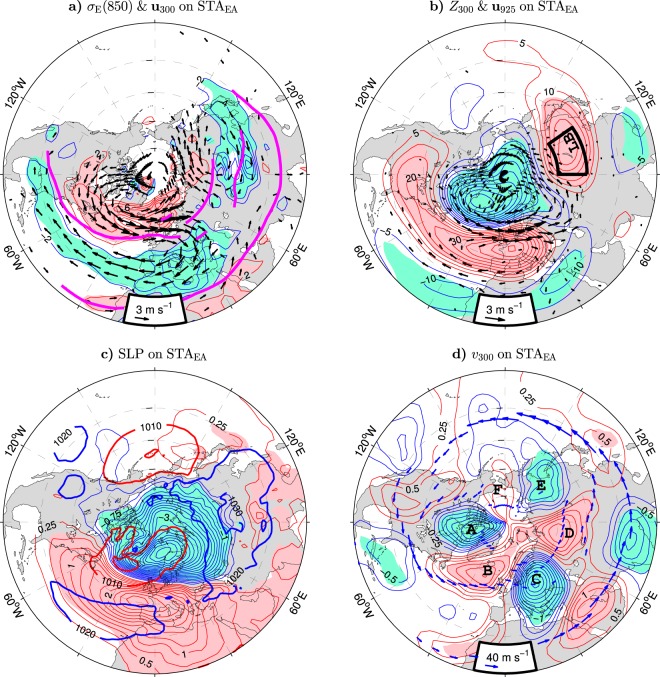
Relation of wintertime tropospheric anomalies to the leading mode of the concurrent storm track activity variations over Eurasia in the ESO period. (**a**–**d**) (Thin contours and color shading), Detrended anomalies of the Eady growth rate at 850 hPa, geopotential height at 300 hPa, sea level pressure and meridional velocity (positive northward) at 300 hPa, respectively, regressed onto the STA_EA_ index (red curve in Fig. 1c). The CI is 2 × 10^−2^ day^−1^, 5 gpm, 0.25 hPa and 0.25 m s^−1^ per unit STA_EA_ index, respectively. The thin contour and shading colors are explained in the caption to Fig. 1. In (**a** and **b**) arrows show detrended anomalies of the horizontal wind velocity (in m s^−1^ per unit STA_EA_ index, subsampled and masked if both components are nonsignificant at the 95% confidence level) at 300 hPa and 925 hPa, respectively. In (**a**) magenta lines depict the wintertime climatology of jet streams in the Atlantic-Eurasian region. In (**b**) the black box delineates the area in eastern Asia over which *Z*_300_ is averaged to obtain the GPH_LB_ index. In (**c**) thick contours show the wintertime climatology of SLP (in hPa). In (**d**) blue arrows depict the wintertime climatology of jet streams in the Northen Hemisphere. The maps were generated by MathWorks MATLAB R2014a with M_Map (http://www.eoas.ubc.ca/rich/map.html).

Anomaly patterns of key wintertime tropospheric variables regressed on the concurrent STA_EA_ index are shown in Fig. 4. The poleward shift of the Atlantic storm track in the positive phase of this index (Fig. 1b) is associated with a poleward migration of the North Atlantic jet. Such migration is indicated by westerly wind anomalies on the northern side of the climatological jet and easterly wind anomalies on its southern side (see Fig. 4a; arrows for significant wind anomalies at 300 hPa and magenta lines for the schematic position of climatological jets in the Atlantic-Eurasian region drawn as tangents to the arrows in Fig. 4d). At its exit to Europe, the anomalous northern stream splits into a poleward and equatorward flow contributing to an anomalous upper-tropospheric polar vortex (cyclonic motion around Greenland) and an anomalous midlatitude anticyclone. A similar anomalous vortex pair appears in the lower troposphere (see arrows in Fig. 4b for significant wind anomalies at 925 hPa). The surface circulation anomalies form an NAO-like pattern that consists of a cyclone around the strengthened, shifted northeastward Icelandic Low and an anticyclone around the strengthened, shifted northeastward Azores High (compare the STA_EA_-related SLP anomalies in Fig. 4c (thin contours and color shading) to the NAO-related SLP anomalies in Supplementary Fig. S2a and see thick contours in Fig. 4c for the SLP climatology).

The zonal wind anomalies in the North Atlantic region are primarily equivalent barotropic, that is, the upper-tropospheric wind anomalies are approximately co-located and in-phase with but stronger than the corresponding near-surface wind anomalies (compare arrows in Fig. 4a,b). The corresponding anomalous vertical shear of the zonal wind should result in anomalous baroclinic instabilities that maintain the storm track anomalies. Increased growth of baroclinic waves should occur in the area of enhanced westerlies on the poleward side of the climatological jet while suppression of the growth of baroclinic waves should occur in the area of decreased westerlies on the equatorward side of the jet. This scenario is supported by the anomaly pattern of the baroclinic instabilities growth rate maximum *σ*_*E*_ (see Eq. (1) in Methods for its definition) at 850 hPa (thin contours and color shading in Fig. 4a).

The anomalous vortices in the Euro-Atlantic sector coexist, during positive polarity of the STA_EA_ index, with an upper-tropospheric anticyclone in Asia that is centered over Lake Baikal (Fig. 4a). This anomalous “Lake Baikal” vortex straddles the climatological polar front jet and weakens a midlatitude jet spreading between the polar and subtropical jets. In the STA_EA_-related anomaly pattern of the geopotential height (GPH) at 300 hPa (*Z*_300_), the vortex appears as a well pronounced ridge (lobe of positive GPH anomalies; see the thin contours and color shading in Fig. 4b). In the subsequent analysis, its phase and strength will be represented by the GPH_LB_ index defined as standardised anomalies of *Z*_300_ averaged around Lake Baikal (LB box in Fig. 4b).

While the configuration of storm track and wind anomalies implies that the upper-tropospheric vortex pair in the Euro-Atlantic sector should be maintained by local eddy-mean flow interactions, the role of synoptic eddies in generating the “Lake Baikal” vortex is less clear. Even though the center of this vortex coincides with a secondary maximum of the STA_EA_-related anomalies of ${\overline{v^{\prime} v^{\prime} }}_{300}$ (compare Figs 4b and 1b), this maximum is fairly weak and not significant at all in the NAO-related pattern of ${\overline{v^{\prime} v^{\prime} }}_{300}$ anomalies (Fig. 1a). The strong relation of the “Lake Baikal” vortex (GPH_LB_ index) to both the leading mode of the storm track variability over Eurasia and the NAO (*r* > 0.7; see Supplementary Table S4) should originate from a teleconnective mechanism. This mechanism may be related to anomalous planetary waves trapped by the Asian jets waveguide^14,45^.

Involvement of quasi-stationary planetary waves in the teleconnection of the upper-tropospheric circulation over northern Asia to the storm track and circulation anomalies in the Euro-Atlantic sector is suggested by the anomaly pattern of the meridional wind *v* at 300 hPa (*v*_300_) regressed onto the STA_EA_ index (thin contours and color shading in Fig. 4d). The pattern exhibits a prominent quasi-zonal wavenumber 3 circumglobal structure of *v*_300_ anomalies with centers of action (marked by letters A-F in Fig. 4d) that are largely confined to the latitudinal band 50°–75°N. Lobes C-E of this high-latitude wavetrain follow the climatological Eurasian polar front jet (see the arrows in Fig. 4d). Lobes D and E are the western and eastern limbs of the “Lake Baikal” vortex, respectively.

### Surface air temperature anomalies

**Figure 5:**
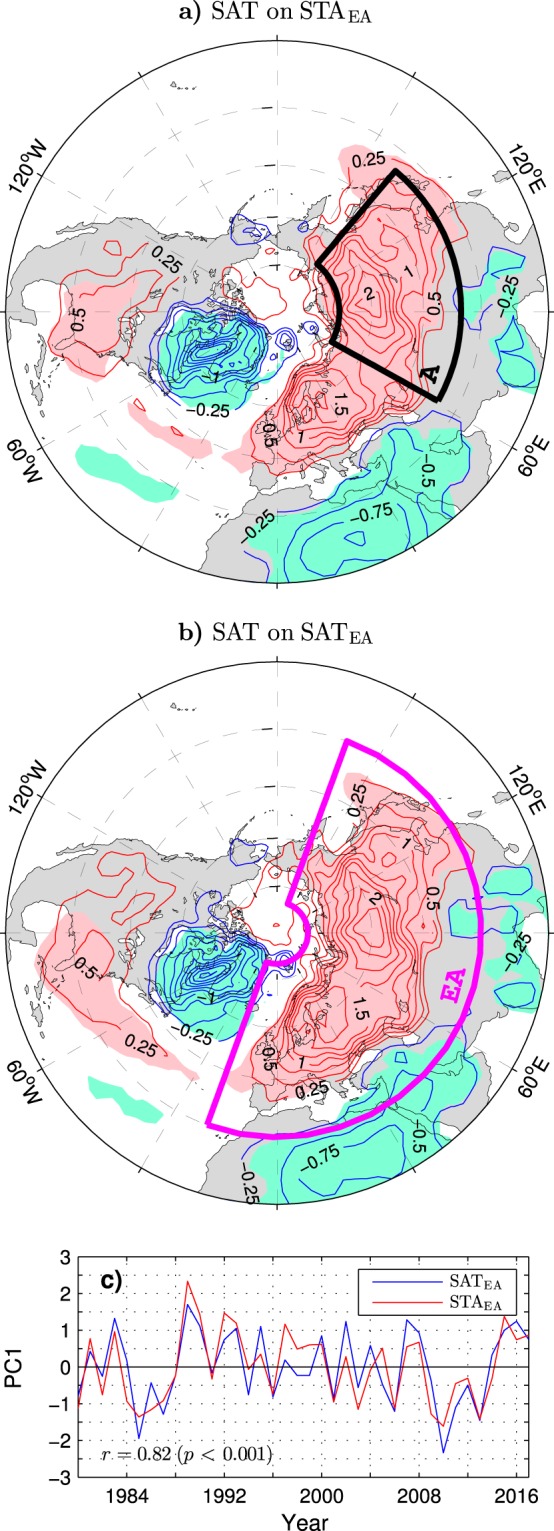
Wintertime anomalies of the surface air temperature associated with leading modes of tropospheric variability over Eurasia in the ESO period. (**a**) Anomalies of SAT regressed onto the STA_EA_ index defined as the PC1 of ${\overline{v^{\prime} v^{\prime} }}_{300}$ variability within the magenta box in Fig. 1b. (**b**) Anomalies of SAT regressed onto the SAT_EA_ index defined as the PC1 of SAT variability over land within the area marked by a magenta box. (**c**) Comparison of the SAT_EA_ (blue curve) and STA_EA_ (red curve) indices (years correspond to the January). All subplots are based on detrended data. In (**a** and **b**) the CI is 0.25 K per the corresponding unit PC1. The contour and shading colors are explained in the caption to Fig. 1. In (**a**) the black box delineates the area in northern Asia over which SAT is averaged to obtain the SAT_A_ index. The maps were generated by MathWorks MATLAB R2014a with M_Map (http://www.eoas.ubc.ca/rich/map.html).

During the positive phase of the STA_EA_ index, the enhanced surface westerlies along the common rim of the strengthened Icelandic Low and Azores High (Fig. 4b,c) efficiently bring warm maritime air onto Europe. At the same time advection of warm air by southerly and southwesterly wind anomalies on the eastern side of the extended Icelandic Low warms western Siberia while advection of cold air by anomalous northerlies and northwesterlies on the western side of this low cools the Labrador Sea/Greenland region. The concurrent advection of relatively cold air by northerly wind anomalies on the eastern side of the extended Azores High cools the eastern Mediterranean/northeastern Africa region. The resulting pattern of SAT anomalies (Fig. 5a) mirrors the corresponding pattern associated with the SAT_EA_ index defined as the PC time series of the leading mode of the wintertime SAT variability in Eurasia north of 30°N (Fig. 5b). Both patterns are very similar to the corresponding NAO-related pattern shown in Supplementary Fig. S2b. Remarkable features of these patterns are distinct, same-sign extremes of comparable magnitude in northern Europe and the central part of northern Asia. The Asian lobe of significant anomalies extends southeastward beyond the area of the direct influence of significant wind anomalies (arrows in Fig. 4b). Therefore, the SAT anomalies in eastern Asia should be maintained by other processes than temperature advection by the anomalous surface winds.

The SAT_EA_ index correlates nearly as highly with the STA_EA_ index (*r* = 0.82; see Fig. 5c for comparison of the time series) as with the NAO index (*r* = 0.86), indicating that air temperature variations in Eurasia are largely controlled by storm track displacements in the Euro-Atlantic sector. The SAT_A_ index defined as standardised anomalies of SAT in Asia north of 35°N (averaged over the black box in Fig. 5a) correlates even higher with the STA_EA_ index (*r* = 0.79) than with the NAO index (*r* = 0.74). This suggests a scenario in which the anomalous upper-tropospheric circulation over Asia (“Lake Baikal” vortex) is a key agent coupling wintertime air temperature anomalies in this area to the storm track variations in the Euro-Atlantic sector.

**Figure 6:**
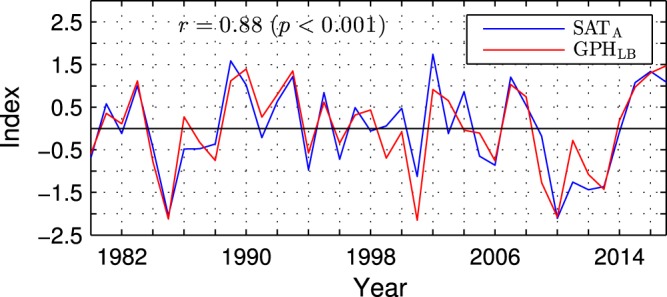
Time series of wintertime anomalies of the surface air temperature and geopotential height at 300 hPa over Asia in the ESO period. The blue curve shows standardised anomalies of SAT averaged over the black box in Fig. 5a (SAT_A_ index). The red curve shows standardised anomalies of GPH averaged over the black box in Fig. 4b (GPH_LB_ index). Both time series are based on detrended data. Years correspond to the January.

The “Lake Baikal” vortex may control anomalous air temperatures in Asia via surface-reaching displacements of isentropic surfaces. During positive polarity of the GPH_LB_ index, the anticyclonic anomaly of the relative (and hence absolute) vorticity in this vortex corresponds to a negative potential vorticity anomaly. Such an anomaly can induce a surface warming by pushing isentropic surfaces downward^49^. Similarly, in the negative phase of the GPH_LB_ index, a positive potential vorticity anomaly corresponding to the cyclonic anomaly of the relative vorticity in the “Lake Baikal” vortex can induce a surface cooling by pulling isentropic surfaces upward in the troposphere. This mechanism was previously proposed as standing behind occasional connections of Asian air temperature anomalies to the NAO on subseasonal timescales^45^. The relation of the winter mean SAT anomalies in Asia to the “Lake Baikal” vortex is very robust. Indeed, the GPH_LB_ index correlates with the SAT_A_ index at 0.88 (see Fig. 6 for comparison of the time series).

## Relation to Pre-Winter Sea Ice Anomalies

### Linkages during the ESO period

**Figure 7:**
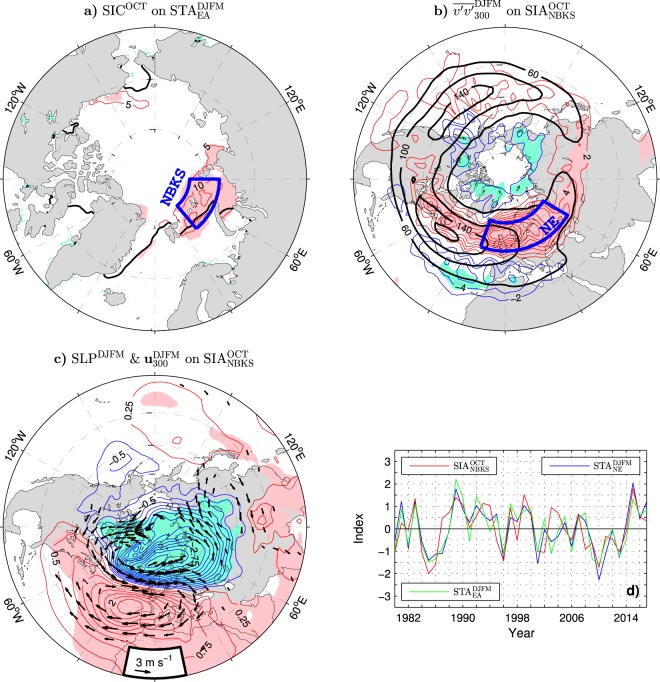
Relation between wintertime tropospheric variability and earlier anomalies of the sea ice concentration in the Arctic during the ESO period. (**a**) (Thin contours and color shading), Detrended anomalies of October SIC regressed onto the following winter STA_EA_ index (red curve Fig. 1c). The CI is 5% per unit STA_EA_ index. Thick black lines show the climatological ice edge in October defined as the 15% SIC contour. (**b**) (Thin contours and color shading), Detrended wintertime anomalies of ${\overline{v^{\prime} v^{\prime} }}_{300}$ regressed onto the previous October SIA_NBKS_ index (SIC integrated over the blue box in (**a**)). The CI is 2 m^2^ s^−2^ per unit SIA_NBKS_ index. Thick black contours represent the wintertime climatology of ${\overline{v^{\prime} v^{\prime} }}_{300}$ (in m^2^ s^−2^). (**c**) (Thin contours and color shading), Detrended wintertime anomalies of SLP regressed onto the previous October SIA_NBKS_ index. Arrows show the corresponding anomalies of the horizontal wind velocity (in m s^−1^ per unit SIA_NBKS_ index, subsampled and masked if both components are nonsignificant at the 95% confidence level) at 300 hPa. (**d**) Time series of the standardised October SIA_NBKS_ index (red curve), wintertime STA_EA_ index (green line) and wintertime STA_NE_ index (blue curve) defined as standardised wintertime anomalies of ${\overline{v^{\prime} v^{\prime} }}_{300}$ averaged over the blue box in (**b**). Years correspond to the January of the wintertime series. In (**a**–**d**) a piecewise linear trend with the breakpoint in 2004 was removed from all sea ice data. The atmospheric data were linearly detrended over the full ESO period. The thin contour and shading colors used in (**a**–**c**) are explained in the caption to Fig. 1. The maps were generated by MathWorks MATLAB R2014a with M_Map (http://www.eoas.ubc.ca/rich/map.html).

A significant relation of wintertime storm track variations over Eurasia to pre-winter sea ice cover anomalies in the northern Barents/Kara Sea is illustrated in Fig. 7a showing the pattern of October SIC anomalies regressed on the following winter STA_EA_ index. October SIC anomalies are shown since a lagged-correlation analysis between the winter STA_EA_ index and indices of the monthly and seasonal mean sea ice area in the northern Barents/Kara Sea (SIA_NBKS_ defined as SIC integrated over the NBKS box in Fig. 7a) revealed that the winter STA_EA_ index correlates higher with October SIA_NBKS_ anomalies than with SIA_NBKS_ anomalies in any other month or season (see Methods and Supplementary Fig. S3). Note also that, prior to the regression and correlation analysis, a continuous piecewise linear trend with the breakpoint in 2004 was removed from the raw SIC and SIA_NBKS_ time series to account for a faster sea ice decline in the second half of the 2000s than earlier^50,51^ (see Supplementary Fig. S4 for the raw time series and the trend removed). This procedure results in higher correlations than obtained when using other detrending methods (see Methods).

The pattern of wintertime anomalies of ${\overline{v^{\prime} v^{\prime} }}_{300}$ regressed on the previous October SIA_NBKS_ index (thin contours and color shading in Fig. 7b) is qualitatively similar to the corresponding patterns associated with the wintertime STA_EA_ and NAO indices (Fig. 1). Its dominant, midlatitude lobe of significant, positive anomalies spreads quasi-zonally north and east of the climatological North Atlantic storm track maximum, from Labrador to the main center of action near the northern tip of the British Isles, and then across northern Eurasia to Japan. In the south, this lobe is accompanied by a relatively weak subtropical lobe of negative anomalies with a center of action west of the Mediterranean Sea. In the north, it is accompanied by a secondary lobe of negative anomalies spreading all around the Arctic Ocean coast. Therefore, an increased October ice cover in the northern Barents/Kara Sea corresponds to an intensification, poleward shift, eastward extension and narrowing of the North Atlantic storm track in the following winter. By analogy, a reduced October ice cover in the northern Barents/Kara Sea corresponds to a weakening, equatorward shift, westward contraction and broadening of the North Atlantic storm track in the following winter. The October SIA_NBKS_ anomalies correlate highly (*r* = 0.70) with the PC time series of the leading wintertime mode of variability in ${\overline{v^{\prime} v^{\prime} }}_{300}$ over Eurasia (STA_EA_ index) and even more highly (*r* = 0.75) with the wintertime anomalies of ${\overline{v^{\prime} v^{\prime} }}_{300}$ averaged around their center of action in northern Europe (NE box in Fig. 7b), denoted as STA_NE_ (see Fig. 7d for comparison of the time series).

**Figure 8:**
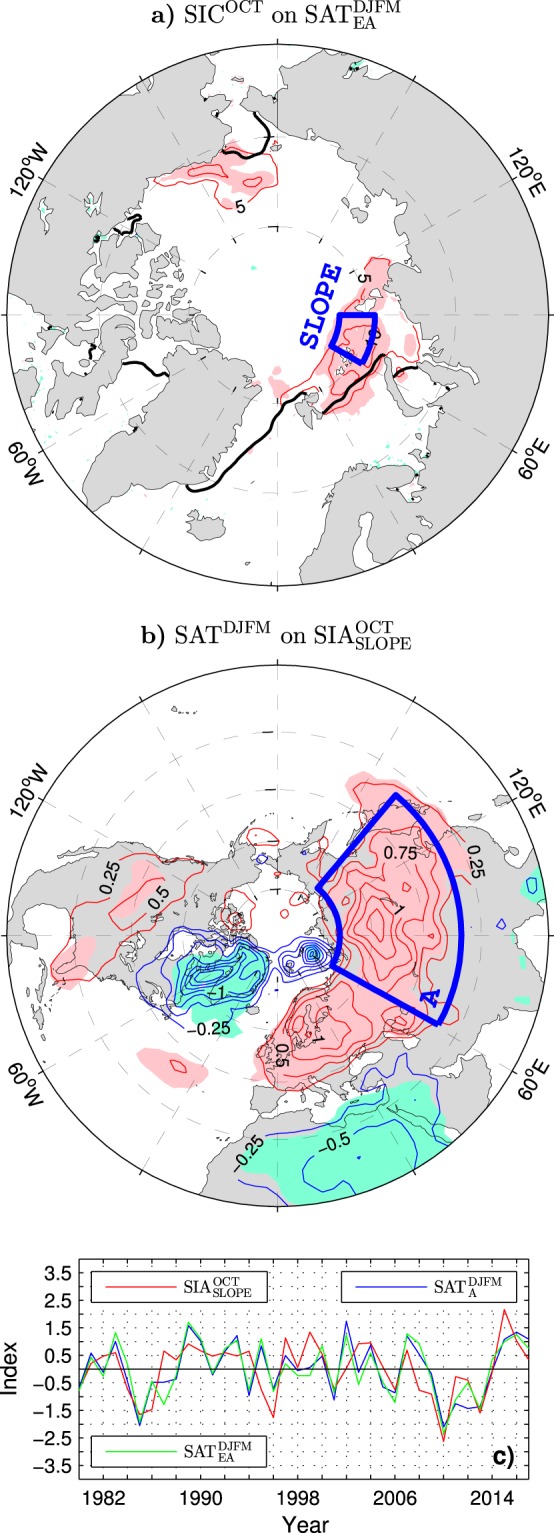
Relation between variations of the wintertime surface air temperature and earlier anomalies of the sea ice concentration in the Arctic during the ESO period. (**a**) (Thin contours and color shading), Detrended anomalies of October SIC regressed onto the following winter SAT_EA_ index (blue curve in Fig. 5c). The CI is 5% per unit SAT_EA_ index. Thick black lines show the climatological ice edge in October defined as the 15% SIC contour. (**b**) Detrended anomalies of the wintertime SAT regressed onto the previous October SIA_SLOPE_ index (SIC integrated over the blue box in (**a**)). The CI is 0.25 K per unit SIA_SLOPE_ index. (**c**) Time series of the standardised October SIA_SLOPE_ index (red curve), wintertime SAT_EA_ index (green line) and wintertime SAT_A_ index (blue curve) defined as standardised wintertime anomalies of SAT averaged over the blue box in (**b**). Years correspond to the January of the wintertime series. In (**a**–**c**) a piecewise linear trend with the breakpoint in 2004 was removed from all sea ice data. The atmospheric data were linearly detrended over the full ESO period. The thin contour and shading colors used in (**a**–**c**) are explained in the caption to Fig. 1. The maps were generated by MathWorks MATLAB R2014a with M_Map (http://www.eoas.ubc.ca/rich/map.html).

Wintertime circulation anomalies associated with the previous October SIA_NBKS_ index form patterns (see Fig. 7c for the anomalies of SLP and wind velocity at 300 hPa) reminiscent of the corresponding NAO-like patterns associated with the winter STA_EA_ index (Fig. 4). The winter NAO index is tied quite strongly (*r* = 0.66) to the October SIA_NBKS_ anomalies and equally strongly (*r* = 0.66) to the corresponding anomalies of the sea ice area over the northern Kara Sea shelf slope alone (SIA_SLOPE_ defined as SIC integrated over the SLOPE box in Fig. 8a). Consequently, the pattern of winter SAT anomalies associated with the October SIA_NBKS_ index (not shown) or October SIA_SLOPE_ index (shown in Fig. 8b) portrays all major NAO thermal features, including a broad lobe of significant warming over Eurasia with distinct European and Asian centers of action during winters after Octobers with above-normal sea ice cover in the Barents/Kara Sea region. The October SIA_SLOPE_ index correlates quite highly (*r* = 0.61) with the PC time series of the leading wintertime mode of SAT variability in Eurasia (SAT_EA_ index) and even more highly (*r* = 0.66) with the wintertime anomalies of SAT averaged over their Asian lobe, that is, the SAT_A_ index (see Fig. 8c for comparison of the time series).

### Recent storm track/sea ice linkage

Wintertime atmospheric variability over Eurasia might have been linked to pre-winter Arctic sea ice cover anomalies more tightly since 2004 (late ESO period) than before 2004 (early ESO period). To show this, Supplementary Table S5 includes correlations of the October SIA_NBKS_ index with PC-based (STA_EA_, NAO and SAT_EA_) and area-averaged (STA_NE_, GPH_LB_ and SAT_A_) indicators of wintertime atmospheric variability calculated for the full ESO period (${{\rm{SIA}}}_{{\rm{NBKS}}}^{{\rm{79}}-16}$ column), its early part (${{\rm{SIA}}}_{{\rm{NBKS}}}^{{\rm{79}}-03}$ column) and its late part (${{\rm{SIA}}}_{{\rm{NBKS}}}^{{\rm{04}}-16}$ column). For instance, the correlation for the area-averaged STA over northern Europe increased from 0.72 in the early ESO period to 0.88 in the late ESO period. The corresponding increase was even larger for the area-averaged upper-tropospheric GPH over Lake Baikal (from 0.48 to 0.75) and the area-averaged SAT in northern Asia (from 0.49 to 0.77).

**Figure 9:**
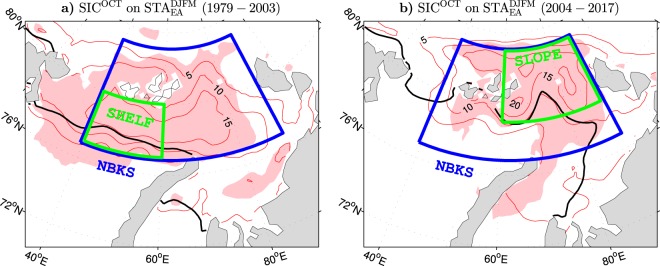
Relation of October anomalies of the sea ice concentration in the Barents/Kara Sea region during the early and late ESO periods to the leading mode of variability in the following winter storm track activity over Eurasia. (**a** and **b**) (Thin contours and color shading), Detrended anomalies of October SIC in the periods 1979–2003 and 2004–2017, respectively, regressed onto the following winter STA_EA_ index (red curve in Fig. 1c). The CI is 5% per unit STA_EA_ index. In (**a** and **b**) thick black lines show the mean ice edge position defined by the 15% contour of October SIC averaged over the early and late ESO periods, respectively. The thin contour and shading colors are explained in the caption to Fig. 1. The maps were generated by MathWorks MATLAB R2014a with M_Map (http://www.eoas.ubc.ca/rich/map.html).

It is noteworthy that sea ice anomalies in the ESO period migrated northeastward through the northern Barents/Kara Sea region with the declining sea ice edge. This is illustrated in Fig. 9a,b showing regression patterns of October SIC anomalies on the part of the winter STA_EA_ index before 2004 and since then, respectively. Before 2004, most significant SIC anomalies appear mainly on the shelf in the northeastern Barents Sea (SHELF box in Fig. 9a) while in the late ESO period most significant SIC anomalies are found in the northern Kara Sea, mainly over the shelf slope (SLOPE box in Fig. 9b). Correlations of the anomalies of SIC integrated over these areas (SIA_SHELF_ and SIA_SLOPE_ indices) with the indicators of wintertime atmospheric variability over Eurasia for the full ESO period together with the corresponding correlations for SIA_SHELF_ before 2004 and SIA_SLOPE_ afterwards are included in Supplementary Table S5. These correlations provide further support for the scenario that the already close link of the wintertime storm tracks over Eurasia and the associated surface climate variability to pre-winter SIC anomalies in the early ESO period may have tightened since the mid-2000s. Specifically, the leading mode of STA variability over Eurasia, which in the early ESO period correlated with SIA_SHELF_ at 0.69, in the late ESO period correlated with SIA_SLOPE_ at 0.86. Similarly, the NAO index was related tightly (*r* = 0.71) to SIA_SHELF_ before 2004 and even more tightly (*r* = 0.81) to SIA_SLOPE_ in the late ESO period. The leading mode of SAT variability in Eurasia, which in the early ESO period was linked to SIA_SHELF_ only moderately (*r* = 0.53), in the late ESO period correlated highly (*r* = 0.81) with SIA_SLOPE_.

The likelihood that the link between atmospheric and sea ice cover anomalies at least as strong as observed in the late ESO period would occur by chance if the strength of this link actually continued unchanged through the entire ESO period is relatively small for all selected indicators of atmospheric variability. This is shown by the results from Monte Carlo simulations (see Methods) reported in Supplementary Table S6. A statistic ${P}_{\ast }$, where the star in the subscript stands for SLOPE, SHELF or NBKS, is defined there as the percentage of Monte Carlo trials in which the correlation between a selected atmospheric index and a given trial SIA (SIA_SLOPE_, SIA_SHELF_ or SIA_NBKS_) index calculated using randomly subsampled data from the early ESO period is not smaller than the correlation between the selected atmospheric index and the SIA_SLOPE_ index in the late ESO period. The likelihood that there was no strengthening of the relationship between atmospheric and sea ice variability in the late ESO period, defined for each atmospheric variable as the maximum *P*_max_ of ${P}_{\ast }$, is given as a bold number in Supplementary Table S6. The most significant increase of the dependence on sea ice anomalies (the smallest *P*_max_) is found for the area-averaged STA over northern Europe and the area-averaged upper-tropospheric GPH over Lake Baikal (*P*_max_ ≈ 2%). The corresponding increase for the leading modes of STA and SAT variability over Eurasia is also quite significant (*P*_max_ ≈ 5%). The increase is less significant for the area-averaged SAT in northern Asia (*P*_max_ = 10%) and the NAO index (*P*_max_ ≈ 20%).

### Empirical forecasts

To demonstrate the predictive value of the lagged relations identified in the preceding sections, empirical forecast models are constructed for the PC-based and area-averaged indicators of the wintertime atmospheric variability for which correlations with sea ice indices of the preceding October are given in Supplementary Table S5. The same sea ice indices or their combinations are used as predictors. The forecast models are based on the liner regression with the so-called leave-1-yr-out cross-validation (see Methods). Results from main forecast experiments are summarised in Table 1, which reports the correlation skill score (CSS) and the proportion of explained variance (PEV) of the forecasts.

**Table 1:** Correlation skill score (CSS) and the proportion of explained variance (PEV) in leave-1-yr-out (L) cross-validation forecasts of wintertime atmospheric anomalies from indices of sea ice variability in the preceding October.

	STA_EA_		STA_NE_		NAO		GPH_LB_		SAT_EA_		SAT_A_	
	CSS	PEV	CSS	PEV	CSS	PEV	CSS	PEV	CSS	PEV	CSS	PEV
${{\rm{L}}}_{{\rm{NBKS}}}^{{\rm{full}}}$	***0***.***67***	0.44	***0***.***72***	0.52	***0***.***61***	0.36	**0**.**50**	0.24	**0**.**49**	0.24	***0***.***55***	0.29
${{\rm{L}}}_{{\rm{SLOPE}}}^{{\rm{full}}}$	***0***.***65***	0.42	***0***.***72***	0.51	***0***.***62***	0.38	***0***.***56***	0.31	***0***.***55***	0.30	***0***.***62***	0.38
${{\rm{L}}}_{{\rm{SLOPE}}}^{{\rm{early}}}$	0.49	0.23	**0**.**56**	0.30	0.41	0.16		0.02		0.03	0.42	0.16
${{\rm{L}}}_{{\rm{SLOPE}}}^{{\rm{late}}}$	**0**.**82**	0.66	***0***.***88***	0.77	**0**.**75**	0.56	**0**.**74**	0.53	0.72	0.51	0.71	0.50
${{\rm{L}}}_{{\rm{SHELF}}}^{{\rm{early}}}$	***0***.***64***	0.41	***0***.***67***	0.45	***0***.***67***	0.44		0.08	0.41	0.15		0.10
${{\rm{L}}}_{{\rm{SHELF}}}^{{\rm{late}}}$		0.21	0.65	0.41		0.06		−0.01		−0.01		0.09
${{\rm{L}}}_{{\rm{joint1}}}^{{\rm{full}}}$	***0***.***73***	0.53	***0***.***76***	0.58	***0***.***73***	0.53	***0***.***56***	0.31	***0***.***59***	0.35	***0***.***56***	0.31
${{\rm{L}}}_{{\rm{joint2}}}^{{\rm{full}}}$	***0***.***74***	0.54	***0***.***76***	0.57	***0***.***71***	0.50	***0***.***57***	0.33	***0***.***62***	0.38	***0***.***63***	0.40

Atmospheric predictands are the PC-based (STA_EA_, NAO and SAT_EA_) and area-averaged (STA_NE_, GPH_LB_ and SAT_A_) indices for which correlations with sea ice indices are given in Supplementary Table S5. Predictors are based on the SIA_NBKS_, SIA_SLOPE_ and SIA_SHELF_ indices representing anomalies of the sea ice concentration integrated over the NBKS box in Fig. 7a, SLOPE box in Fig. 9b and SHELF box in Fig. 9a. Prior to the forecast experiments, a continuous piecewise linear trend with the breakpoint in 2004 was removed from the SIA time series. The atmospheric time series were linearly detrended over the full ESO period (winters 1980–2017, years of the January). The forecasts are performed for either the full ESO period or its early (winters 1980–2004) and late (winters 2005–2017) parts (as indicated in the superscript of L) using the SIA index from the region indicated in the subscript of L. The predictor in forecasts ${{\rm{L}}}_{{\rm{joint1}}}^{{\rm{full}}}$ is the SIA_joint1_ index (blue curve in Supplementary Fig. S5b) obtained by merging the time series of the SIA_SHELF_ anomalies from the early ESO period with the SIA_SLOPE_ anomalies from the late ESO period. The predictor in forecasts ${{\rm{L}}}_{{\rm{joint2}}}^{{\rm{full}}}$ is the SIA_joint2_ index (red curve in Supplementary Fig. S5b) obtained by merging the time series of the SIA_SHELF_ anomalies from the period 1979–1994 with the SIA_SLOPE_ anomalies from the period 1995–2016. The CSS values are given if significant at the 95% confidence level. The CSS values significant at the 99% (99.9%) level are in boldface (boldface and italic).

The first two experiments (denoted as ${{\rm{L}}}_{{\rm{NBKS}}}^{{\rm{full}}}$ and ${{\rm{L}}}_{{\rm{SLOPE}}}^{{\rm{full}}}$ in Table 1) are performed for the full ESO period using the sea ice cover anomalies in, respectively, the entire northern Barens/Kara Sea region (SIA_NBKS_ index, red curve in Fig. 7d) and its northeastern subdomain (SIA_SLOPE_ index, red curve in Fig. 8c) as predictors. The skill scores from these experiments indicate that the SIA_NBKS_ and SIA_SLOPE_ indices are, in practice, equally good predictors (differences in PEV ≤ 0.02) of the atmospheric variables that are either based on data from the Euro-Atlantic sector (STA_NE_ index) or associated with major centers of action located in this sector (STA_EA_ and NAO indices). However, in the forecasts of the atmospheric variables that are either based on data from the Asian sector (GPH_LB_ and SAT_A_ indices) or associated with a major center of action located in this sector (SAT_EA_ index), the SIA_SLOPE_ index outperforms the SIA_NBKS_ index as the predictor (differences in PEV > 0.05). In the ${{\rm{L}}}_{{\rm{SLOPE}}}^{{\rm{full}}}$ experiment, the PEV scores range from 0.3 for the leading mode of air temperature variability in Eurasia (SAT_EA_ index) to about 0.5 for the area-averaged storm track anomalies over northern Europe (STA_NE_ index).

A recent increase in the skill of the prediction of all selected atmospheric variables from indices of sea ice variability is demonstrated by forecast experiments performed separately for the early and late ESO periods. In these experiments, the SIA_SLOPE_ index and the sea ice cover anomalies in the southwestern subdomain of the northern Barents/Kara Sea region (SIA_SHELF_ index, blue curve in Supplementary Fig. S5a) are used as predictors. In Table 1, these experiments are denoted as ${{\rm{L}}}_{{\rm{SLOPE}}}^{{\rm{early}}}$, ${{\rm{L}}}_{{\rm{SLOPE}}}^{{\rm{late}}}$, ${{\rm{L}}}_{{\rm{SHELF}}}^{{\rm{early}}}$ and ${{\rm{L}}}_{{\rm{SHELF}}}^{{\rm{late}}}$ (note that the CSS values are given if they are significant at the 95% or higher confidence level). In the late ESO period, all forecasts with SIA_SLOPE_ have significant CSS values and high (≥ 0.5) PEV scores. The PEV score is exceptionally high (0.77) for the STA anomalies over northern Europe and is also very high (0.66) for the leading mode of the storm track variability over Eurasia (STA_EA_ index). In the same period, the forecasts with SIA_SHELF_ have a significant CSS value and a relatively high (> 0.4) PEV score only for the STA_NE_ index. In contrast, in the early ESO period, the forecasts for all atmospheric indices but the area-averaged SAT anomalies in northern Asia (SAT_A_ index) have higher scores with the SIA_SHELF_ than SIA_SLOPE_ as the predictor. However, in that period, relatively high (> 0.4) PEV scores are obtained only for the storm track indices (STA_NE_ and STA_EA_) and the NAO index.

Since the SIA_SHELF_ index is generally a better predictor in the early ESO period while the SIA_SLOPE_ index is a better predictor in the late ESO period, an additional forecast experiment (denoted as ${{\rm{L}}}_{{\rm{joint1}}}^{{\rm{full}}}$ in Table 1) is performed for the full ESO period using the SIA_joint1_ index as the predictor. This predictor (blue curve in Supplementary Fig. S5b) is obtained by merging the time series of SIA_SHELF_ anomalies from the early ESO period with the SIA_SLOPE_ anomalies from the late ESO period. The forecasts in the ${{\rm{L}}}_{{\rm{joint1}}}^{{\rm{full}}}$ experiment outperform the forecasts in the ${{\rm{L}}}_{{\rm{SLOPE}}}^{{\rm{full}}}$ experiment except for the SAT anomalies in northern Asia and for the upper-tropospheric geopotential height anomalies over Lake Baikal (GPH_LB_ index). The largest gain in PEV (from 0.38 to 0.53) is obtained for the NAO index. The predictability is also considerably improved for the storm track indices, which in the ${{\rm{L}}}_{{\rm{joint1}}}^{{\rm{full}}}$ experiment achieve PEV scores of 0.58 (STA_NE_ index) and 0.53 (STA_EA_ index). The improvement is slightly weaker for the leading mode of SAT variability in Eurasia. The predictability of this mode is, however, slightly higher when the time series of SIA_SHELF_ and SIA_SLOPE_ anomalies are merged in the mid 1990s instead of the mid 2000s. Using the SIA_joint2_ index (SIA_SHELF_ anomalies to 1994 and SIA_SLOPE_ anomalies afterwards; see the red curve in Supplementary Fig. S5b) as the predictor (${{\rm{L}}}_{{\rm{joint2}}}^{{\rm{full}}}$ experiment in Table 1) yields relatively high forecast skill scores (PEV ≈ 0.4 and CSS ≥ 0.6) for both SAT indices (SAT_EA_ and SAT_A_). For other atmospheric variables, the forecast skill scores are practically as high in the ${{\rm{L}}}_{{\rm{joint2}}}^{{\rm{full}}}$ experiment as in the ${{\rm{L}}}_{{\rm{joint1}}}^{{\rm{full}}}$ experiment (PEV differences ≤ 0.02). The highest CSS values are obtained for the STA anomalies over northern Europe (0.76 in both ${{\rm{L}}}_{{\rm{joint2}}}^{{\rm{full}}}$ and ${{\rm{L}}}_{{\rm{joint1}}}^{{\rm{full}}}$), the leading mode of STA variability over Eurasia (0.74 in ${{\rm{L}}}_{{\rm{joint2}}}^{{\rm{full}}}$) and the NAO index (0.73 in ${{\rm{L}}}_{{\rm{joint1}}}^{{\rm{full}}}$).

**Figure 10:**
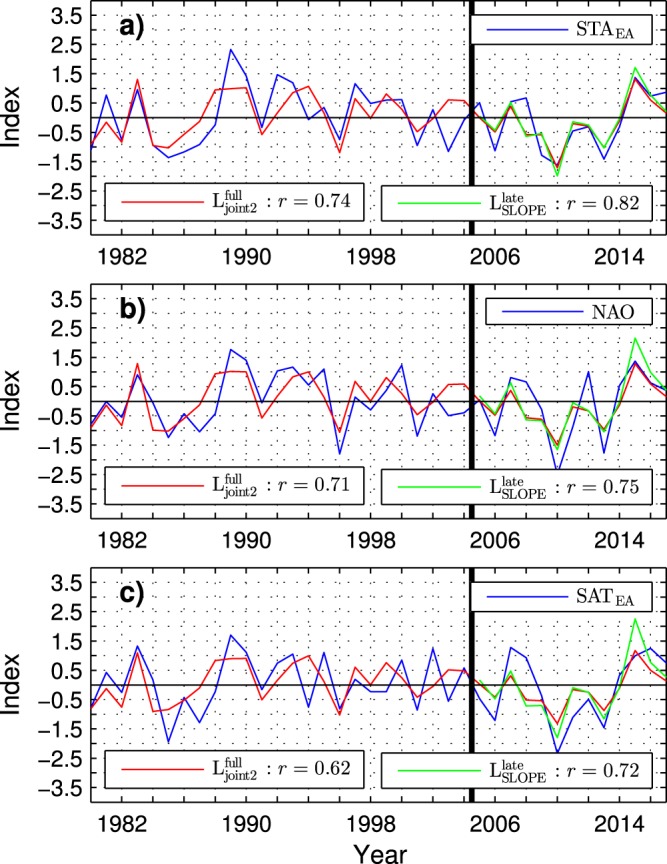
Time series of observed and predicted wintertime tropospheric anomalies during the ESO period. (**a**) PC1 of the observed storm track activity variations over Eurasia (blue contour), its leave-1-yr-out prediction from the preceding October SIA_joint2_ index of the sea ice cover variability in the northern Barents/Kara Sea region during the full ESO period (red curve) and from the preceding October SIA_SLOPE_ index of sea ice cover variability in the northern Kara Sea region during the late ESO period (green curve). See Methods or caption to Table 1 for the definition of the SIA indices. (**b**) As **a** except for the NAO index. (**c**) As **a** except for the PC1 of surface air temperature variations in Eurasia. The vertical black line separates the early and late ESO periods.

To investigate predictability of particular events, Fig. 10 compares the PC-based indices of the wintertime atmospheric variability (blue lines) to their forecasts from the preceding October ${{\rm{SIA}}}_{{\rm{joint2}}}^{{\rm{full}}}$ index in the full ESO period (red lines) and from the preceding October ${{\rm{SIA}}}_{{\rm{SLOPE}}}^{{\rm{late}}}$ index in the late ESO period (green lines). In the late ESO period, the sea ice indices predict the highest negative and positive value of the STA_EA_ index for winters 2010 and 2015, respectively (Fig. 10a). They also predict extreme negative and positive values of the NAO index (Fig. 10b) and the SAT_EA_ index (Fig. 10c) for the same winters. These forecasts fit very well the observations. The observed STA_EA_ and NAO indices indeed exhibit absolute negative and positive extremes during the ESO period in winters 2010 and 2015, respectively. Similarly, the SAT_EA_ index shows a minimum exceeding 2 standard deviations in 2010 and a high positive value (about 1 standard deviation) in 2015. The prediction of a third strongest event in the late ESO period (a secondary minimum) for the winter 2013 is also verified by the observed STA_EA_, NAO and SAT_EA_ indices. To some extent, the sea ice anomalies are skillful predictors of atmospheric extremes also in the early ESO period. For instance, the magnitude of the forecasted STA_EA_ and NAO indices is equal to or exceeds 1 standard deviation in six winters (1983, 1985, 1989, 1990, 1994 and 1996) of this period. In five (83%) of these winters (all but the 1994 winter), the observed STA_EA_, NAO and SAT_EA_ indices have the correct sign and a magnitude equal to or greater than about 0.7 standard deviations.

Since the NAO index is a benchmark index for verification of seasonal climate prediction systems, additional forecast experiments are performed for this index to test the forecast sensitivity to the definition of the index, to the length of the winter season and to the way of detrending the sea ice data. These experiments are summarised in Supplementary Table S7 and discussed in Methods. They show, in particular, that the forecast skill scores are higher for the NAO indices computed from SLP data averaged over the DJFM season than shorter timespans and that the piecewise linear detrending of the sea ice data results in considerably higher forecast skill scores than the simple linear detrending of these data. For instance, while the CSS value of the forecast of a domain-based DJFM-mean NAO index in the ESO period from the piecewise detrended SIA_joint1_ index is 0.72, the CSS value of the forecast of the corresponding December-January-February (DJF) mean NAO index from the analogue of the SIA_joint1_ index based on the time series linearly detrended over the full ESO period is only 0.54. The latter value is very close to the anomaly correlation coefficient of 0.51 between the same (domain-based DJF-mean) observed NAO index in the period 1980–2015 and its leave-1-yr-out empirical prediction from a PC-based index of the Arctic sea ice cover variability in October reported in ref.^41^.

## Discussion

Previous studies on dominant regional and hemispheric modes of storm track variability, based on time series covering approximately the second half of the 20th century, have emphasised a link of the NAO and its hemispheric generalisation, the so-called Arctic Oscillation^52^, to “pulsating” modes of STA variability characterised by a variable amplitude of anomalies but negligible spatial displacements^46,53^. Here it is shown that, in the ESO period (1979–2017), the wintertime NAO index is significantly related to a “spatially shifting” mode of the North Atlantic storm track variability. In the positive phase of its PC time series, this mode corresponds to a northward shift and eastward expansion of the North Atlantic storm track. This mode is related to the NAO index even more tightly if computed over extratropical Eurasia instead of the North Atlantic region. The PC time series of its “Eurasian” version (STA_EA_ index) explains as much as about 70% of the NAO variance.

At the surface, the STA_EA_-related circulation anomaly mirrors the NAO-related changes in the strength of the Icelandic Low and Azores High. In the upper troposphere, it has a wide horizontal structure, with vortices in the Euro-Atlantic sector accompanied by a vortex over Asia centered at Lake Baikal. This “Lake Baikal” vortex is, as indicated by the STA_EA_-related upper-tropospheric anomalies of the meridional wind, linked to the storm track variations in the Euro-Atlantic sector via a high-latitude, wavenumber 3 circumglobal quasi-stationary planetary wave. The Eurasian lobes of this wave are trapped by the polar front jet. A similar high-latitude circumglobal pattern was previously identified by an EOF analysis of the DJF mean meridional wind at 300 hPa during the period 1958–2011 from the NCEP/NCAR reanalysis and its prominence supported by climate models^54^. Interactions of the North Atlantic storm track with the wavenumber 3 wave may be responsible for (or contribute to) coherent wintertime SAT variations from the Atlantic to the Pacific coast of northern Eurasia. Such a scenario is compatible with a strong link of the leading mode of SAT variability in extratropical Eurasia (SAT_EA_ index) to the STA_EA_ index (more than 65% of the variance explained). It is also supported by a strong link of the area-averaged SAT anomalies in Asia north of 35°N (SAT_A_ index) to the GPH_LB_ index representing variability of the “Lake Baikal” vortex (more than 75% of the variance explained).

The present study also reveals a strong linkage of the wintertime storm track variability over Eurasia to the previous October sea ice cover anomalies in the Barents/Kara Sea region and shows that this linkage might have tightened in recent years. Before the acceleration of the sea ice decline in the mid 2000s (early ESO period), 48% of the STA_EA_ variance is accounted for by area-averaged SIC anomalies over the northeastern Barents Sea shelf (SIA_SHELF_ index). In the period since 2004 (late ESO period), 74% of the STA_EA_ variance is explained by area-averaged SIC anomalies over the northern Kara Sea shelf slope (SIA_SLOPE_ index). For that period, the leave-1-yr-out cross-validated predictions of the wintertime STA_EA_ and NAO indices from the October SIA_SLOPE_ index yield forecast correlation skill scores as high as 0.82 and 0.75, respectively. The corresponding skill scores of the STA_EA_ and NAO predictions for the entire ESO period from the SIA_SHELF_ and SIA_SLOPE_ indices merged in 2003/2004 are also high (0.73 for both predictands). The October sea ice cover anomalies in the Barents/Kara Sea region are also found to be a useful predictor of the wintertime SAT variations in Eurasia (about 40% of the cross-validated variance of the SAT_EA_ and SAT_A_ indices explained during the entire ESO period).

The NAO prediction skill scores obtained here are comparable to the NAO prediction skill scores achieved in the current multi-predictor empirical forecast systems and the state-of-the-art dynamical models. Recent benchmarks in the NAO prediction from multisystem ensemble mean dynamical forecasts are anomaly correlation coefficients between an observed and a predicted DJF mean NAO index of 0.65 for the period 1983–2011 and 0.85 for the period 1997–2011^36^. The benchmark from a leave-1-yr-out empirical forecast of a DJF mean NAO index in the period 1980–2015 based on a multiple linear regression technique is an anomaly correlation coefficient of 0.76^41^. The anomaly correlation coefficient of the corresponding NAO forecast from a single, best predictor (EOF-derived time series of October Arctic SIC anomalies with a center of action in the Barents/Kara Sea region)^41^ is considerably lower (by more than 0.2) than the correlation skill score obtained here for the NAO prediction form the sea ice cover anomalies in the Barents/Kara Sea region over approximately the same period. The higher skill of the NAO prediction in the present study than in ref.^41^ results not only from a different sea ice index used as the predictor but also from a different strategy of data detrending (piecewise linear instead of linear) and from the extended winter season (DJFM instead of DJF).

The high predictability of the wintertime STA_EA_/NAO variations strongly suggests that these variations represent a forced mode of atmospheric variability or a coupled mode of variability in the ocean/cryosphere/atmosphere system. Arctic sea ice cover anomalies may trigger storm track/mean flow interactions either directly via the effect of changes in static stability of the lower troposphere on the growth of baroclinic waves^55^ or via excitation of planetary waves that propagate through the troposphere^56^ or into the stratosphere^57,58^. The possible recent tightening of the Arctic sea ice/Eurasian climate linkage might have been related to an increased impact of Atlantic water inflows on the sea-ice loss along the Barents/Kara Sea shelf slope^59^ and subsequent reorganisation of Arctic sources of atmospheric waves. In any case, the reliability of climate predictions and projections by dynamic model systems should critically depend on the ability of these systems to correctly represent ocean/sea-ice/air interactions in the Arctic and their impact on storm tracks.

## Methods

### Atmospheric reanalysis data and derived fields

Winter (DJFM) mean atmospheric fields and their climatology (long-term averages) in the ESO period (1980–2017, years of the January) are constructed using daily mean fields of the meridional wind *v* at 300 hPa and monthly mean fields of several other variables from the National Centers for Environmental Prediction/National Center for Atmospheric Research (NCEP/NCAR) reanalysis^60^. These data, with a longitude-latitude resolution of 2.5° × 2.5°^60^, are downloaded from http://www.esrl.noaa.gov/psd/. The daily data of *v* are used to calculate the DJFM variance of the synoptic meridional wind at 300 hPa, denoted as ${\overline{v^{\prime} v^{\prime} }}_{300}$, where the overbar and prime stand for seasonal averaging and band-pass filtering, respectively. A 2–6 day band-pass filter with weights [−1 −3 −5 18 −5 −3 −1]/24 is employed to calculate *v*′^61^.

The monthly fields include the sea level pressure (SLP), surface air temperature (SAT), geopotential height (GPH) at 300 hPa (*Z*_300_), and meridional and zonal components of the wind velocity **u** at 925 hPa (**u**_925_) and 300 hPa (**u**_300_). In addition, the potential temperature *θ* and wind velocity at 925, 850 and 700 hPa are used to calculate the baroclinic instabilities growth rate maximum^62^ at 850 hPa. This maximum, also known as the Eady growth rate^12^, is defined as

1 $${\sigma }_{E}=0.31\,|\frac{f}{N}\frac{{\rm{\partial }}{\bf{u}}}{{\rm{\partial }}z}|,$$

where *f* is the Coriolis parameter, *N* = [(*g*/*θ*)(∂*θ*/∂*z*)]^1/2^ is the buoyancy frequency, *g* is acceleration due to gravity and *z* is the log-pressure coordinate.

To illustrate the geographical location of jet streams, a refined version of the procedure used in ref.^19^ is applied. At each longitude in the NH extratropics, up to three grid points (latitudes) are found where the climatological wintertime wind speed at 500 hPa during the ESO period reaches a local maximum. Climatological wind vectors at these points are plotted in Figs 1b and 4d. While the selection of grid points is made using wind data at 500 hPa where separated wind cores are more clearly identified, the wind vectors are plotted at 300 hPa where the wind speed is higher.

### Empirical Orthogonal Function analysis

The EOF analysis^63^ is employed to identify recurrent, spatially-coherent changes of winter mean atmospheric (STA, SLP and SAT) fields in the NH extratropics. The EOF decomposition of the wintertime STA and SLP fields is performed for the North Atlantic region (20°–80°N, 90°W–40°E; NA box in Fig. 1a). EOF modes of the STA variability are also derived for a “circumglobal” set of 130°-wide overlapping sectors in the latitudinal band from 30° to 80°N, including the Eurasian sector (30°–80°N, 10°–140°E; EA box in Fig. 1b). The EOF decomposition of the wintertime SAT field is carried out for Eurasia north of 30°N (land within the EA box in Fig. 5b).

The EOF technique extracts from the temporal covariance matrix of a selected field *F* in a given region a set of leading modes of variability consisting of spatial patterns (eigenvectors) and corresponding temporal coefficients, referred to as principal component (PC) time series, that account for a substantial fraction of the total variance of *F* in the given region. To account for different areas represented at each grid point, the anomalies are weighted by the square root of the cosine of latitude prior to constructing the covariance matrix. The regional eigenvectors are not used directly. Instead, the anomalies of *F* and other selected fields in the entire NH extratropics are regressed onto standardised PCs, so that the resulting maps represent informative anomaly patterns (in units of the regressed field) associated with one standard deviation of the reference indices. To focus on the interannual variability, the covariance matrix is built from anomalies of *F* obtained by removing the local linear trend at each grid point. For comparison’s sake, EOF decompositions of the STA and SLP fields are also carried out using nondetrended data (anomalies from the wintertime climatology).

The “rule of thumb” is used to assesses the uniqueness of EOF modes through assumptions of uncertainty on eigenvalues^47^. The uncertainty on the eigenvalue *λ*_*k*_ of the *k*-th mode is ${\rm{\Delta }}{\lambda }_{k}=\sqrt{2/N}{\lambda }_{k}$, where *N* is the number of independent samples (assumed equal to the length of the time series). Let *j* be the mode with the eigenvalue *λ*_*j*_ closest to *λ*_*k*_ and *s* = (Δ*λ*_*k*_ + Δ*λ*_*j*_)/|*λ*_*k*_ − *λ*_*j*_| denote the ratio of the sum of the uncertainty on modes *k* and *j* and the difference of their eigenvalues. If this ratio is greater than one, the “error bars” on *λ*_*k*_ and *λ*_*j*_ overlap indicating a possible mixture of signals between modes *k* and *j*.

Basic information on selected EOF modes, such as the fractional variance of the anomalies in the given region accounted for by the selected mode and significance *s* of this mode, is provided in Supplementary Table S2 for nondetrended data and in Supplementary Table S3 for detrended data.

### Basic atmospheric indices

Three PC-based indices and three indices based on area-averaged quantities are selected for the main analysis of the interannual variability in wintertime atmospheric fields during the ESO period. All these indices are constructed using detrended data. Storm track variations are characterised by the STA_EA_ and STA_NE_ indices. The STA_EA_ index is defined as the PC time series of the leading mode of ${\overline{v^{\prime} v^{\prime} }}_{300}$ variability (PC1_STA–EA_) over the Eurasian sector of the NH extratropics (EA box in Fig. 1b). The STA_EA_ mode explains 28% of the total variance of the wintertime STA anomalies in this sector. The STA_NE_ index represents anomalies of ${\overline{v^{\prime} v^{\prime} }}_{300}$ averaged over northern Europe (50°–65°N, 20°W–60°E; NE box in Fig. 7b). Variations of tropospheric circulation are characterised by the NAO and GPH_LB_ indices. The basic NAO index is defined as the PC time series of the leading mode of SLP variability (PC1_SLP–NA_) in the North Atlantic region (NA box in Fig. 1a). The NAO mode accounts for 52% of the total variance of the wintertime SLP anomalies in this region. The GPH_LB_ index represents anomalies of the geopotential height at 300 hPa averaged over the Lake Baikal area (45°–60°N, 90°–125°E; LB box in Fig. 4b). Air temperature variations are characterised by the SAT_EA_ and SAT_A_ indices. The SAT_EA_ index is defined as the PC time series of the leading mode of SAT variability (PC1_SAT–EA_) over Eurasia (land within the EA box in Fig. 5b). The SAT_EA_ mode explains 48% of the total variance of the wintertime SAT anomalies in this region. The SAT_A_ index represents anomalies of the surface air temperature averaged over northern Asia (35°–70°N, 60°–140°E; A box in Fig. 5a). Sampling errors of the STA_EA_, NAO and SAT_EA_ modes are small enough (*s* < 1; see Supplementary Table S3) to consider them independent from higher order modes. Their time series (PC1_STA–EA_, PC1_SLP–NA_ and PC1_SAT–EA_) are strongly interrelated (see the correlations in Supplementary Table S3) and their relation to the selected area-averaged indices (STA_NE_, GPH_LB_ and SAT_A_) is highly significant (*p* < 0.001; see Supplementary Table S4).

To check the robustness of some NAO-related results to the definition of the NAO index, three other wintertime NAO indices are used in addition to the PC-based wintertime NAO index. The first one is a domain-based NAO index defined as the difference between the SLP anomalies averaged over [25°–50°N, 50°W–10°E] and [55°–85°N, 40°W–20°E] boxes^41^. The second one is a latitude-based NAO index defined as the difference of normalised SLP between two latitude zones (35° and 65°N)^64^. The SLP in these zones is averaged over the longitudes of 80°W–30°E. The third one is the commonly used station-based NAO index representing the difference of normalised SLP between Lisbon, Portugal and Stykkisholmur/Reykjavik, Iceland^16,65^. The domain-based and latitude-based NAO indices are computed using the SLP data from the NCEP/NCAR reanalysis.

### Sea ice data and indices

The analysis of the relation of atmospheric variability to Arctic sea ice cover anomalies is carried out for the ESO period, for which high-quality SIC datasets derived from satellite observations are available. Here, monthly mean SIC fields in the Northern Hemisphere from NOAA’s National Snow and Ice Data Center (NSIDC)^66^, available at http://nsidc.org/, are used. The fields are generated from brightness temperature data derived from Nimbus-7 Scanning Multichannel Microwave Radiometer (SMMR) and Defense Meteorological Satellite Program (DMSP) -F8, -F11 and -F13 Special Sensor Microwave/Imager (SSM/I) and DMSP-F17 Special Sensor Microwave Imager/Sounder (SSMIS) radiances at a grid cell size of 25 km by 25 km. Both monthly and seasonal (4-month) mean fields are initially considered.

A continuous piecewise linear trend with the breakpoint in 2004 is removed from the raw time series of the local SIC and the sea ice area (SIA; integrated SIC) in selected regions to account for an acceleration of the Arctic sea ice decline in the 2000s. This acceleration is illustrated in Supplementary Fig. S4 showing two time series of pre-winter SIA in the northern Barents/Kara Sea region (77°–83°N, 40°–90°E; NBKS box in Fig. 7a), denoted as SIA_NBKS_, together with the corresponding piecewise linear trends. The time series are shown for October (blue lines) and early autumn (August-to-November, ASON) (red lines). These are the month and season for which indices of SIA_NBKS_ (defined as standardised departures of the raw SIA_NBKS_ from its piecewise linear trend for the given month or season) are most significantly correlated with the wintertime STA_EA_ index (see the time development of lagged correlations in Supplementary Fig. S3). As the correlation for the October SIA_NBKS_ index (*r* = 0.70; red curve at lag -2 months) is higher than the correlation for the ASON SIA_NBKS_ index (*r* = 0.65; blue curve at lag -4 months), the October SIA_NBKS_ index is selected for the analysis in the main text. Its correlation with the STA_EA_ index is lower by 0.08 (0.04) if it is based on departures from the linear (third order polynomial) trend over the full ESO period instead of departures from the piecewise linear trend.

To emphasise some features of SIC variability, two additional October SIA indices are constructed, one (SIA_SHELF_) for the northeastern Barents Sea shelf region (77°–80°N, 40°–60°E; SHELF box in Fig. 9a) and the other one (SIA_SLOPE_) for the northern Kara Sea shelf slope region (79°–83°N, 60°–90°E; SLOPE box in Fig. 9b). These indices are compared in Supplementary Fig. S5a. Correlations of all October SIA indices with selected indices of wintertime atmospheric variability in the entire ESO period and its early (1979–2003, years of the SIA indices) and late (2004–2016) parts are given in Supplementary Table S5 and discussed in the main text. All correlations are obtained for the time series from which the linear trend (atmospheric data) or piecewise linear trend (SIA data) is removed.

### Correlation analysis

Correlation analysis is performed to investigate relationships between the reference indices and the regressed fields and between different indices. The statistical significance (or *p*-value) of the correlation coefficient *r* is estimated using a two-tailed Student’s *t* test. All correlations are computed using detrended data unless stated differently. To account for the serial correlation in the time series, the statistical significance tests are carried out with an effective sample size estimated using equation (31) from ref. ^67^. The 95% confidence level is used as a threshold for marking significant correlations in all plots shown. All correlations given in the text for the ESO period are significant at least at the 99.9% confidence level (*p* ≤ 0.001) and for shorter periods at least at the 95% confidence level (*p* ≤ 0.05).

Monte Carlo simulations are carried out to check whether the recent increase of correlations between indices of wintertime atmospheric variability and sea ice anomalies during the preceding autumn suggested by the data from Supplementary Table S5 is statistically significant. To this end, first, a given atmospheric index, say *I*, is linearly detrended over the entire ESO period while the sea ice indices (SIA_SLOPE_, SIA_SHELF_ and SIA_NBKS_) are piecewise linearly detrended with the breakpoint in 2004 separating the early and late ESO subperiods. Subsequently, 10 000 realisations of 13 pairs of data of *I* and the given SIA index are generated by randomly subsampling the 25 years of detrended anomalies from the early ESO period. Finally, it is tested in how many of these realisations the correlation between the given SIA index exceeds or is equal to the correlation between the 13-year-long time series of the detrended anomalies of *I* and SIA_SLOPE_ in the late ESO period. The result of this test for six atmospheric variables is presented in Supplementary Table S6. The SIA_SLOPE_ anomalies are selected as the tested sea ice index for the late ESO period since, in that period, they correlate with all selected atmospheric variables higher than the anomalies of SIA_SHELF_ or SIA_NBKS_ do.

### Forecast experiments

Empirical forecast models are constructed for six wintertime atmospheric variables (STA_EA_, STA_NE_, NAO, GPH_LB_, SAT_EA_ and SAT_A_) for which correlations with sea ice anomalies in the preceding October are given in Supplementary Table S5. Basic predictors are the SIA_NBKS_, SIA_SLOPE_ and SIA_SHELF_ indices. Prior to forecast experiments, a continuous piecewise linear trend with the breakpoint separating the early and late ESO periods is removed from these indices while the atmospheric variables are linearly detrended over the entire ESO period. Two additional predictors, SIA_joint1_ and SIA_joint2_ (shown in Supplementary Fig. S5b), are constructed. The SIA_joint1_ predictor is obtained be merging the time series of the SIA_SHELF_ anomalies from the early ESO period (1979–2003) with the time series of SIA_SLOPE_ anomalies from the late ESO period (2004–2016). The SIA_joint2_ predictor is obtained be merging the SIA_SHELF_ and SIA_SLOPE_ anomalies in 1994/1995 instead of 2003/2004.

The forecast experiments are carried out using the standard linear regression method with the leave-1-yr-out cross-validation scheme^68^. Here, application of this scheme is justified by a relatively low (0.05–0.30) lag-1 autocorrelation of all predictors and predictands used in the forecasts. In the leave-1-yr-out experiments, the training data are strictly separated from the testing data. One year is first excluded from *K* years of data. The forecast model is then trained on the data from the remaining years and tested on the excluded data. The procedure is repeated for each year, providing *K* test results from which two forecast skill scores are estimated. The first one is the correlation skill score (CSS) defined as the correlation coefficient between the forecasts *F* (predicted values) and their targets *T* (predictand values). The second one is the proportion of explained variance (PEV) defined as PEV = 1 − *Var*(*F* − *T*)/*Var*(*T*), where *Var* stands for the variance^63^. PEV is positive if the regression model is better than the climate reference model (*F* = 0 in our case). It becomes unity for a perfect model, that is, when *Var*(*F* − *T*) = 0, and minus unity for a random forecast. The CSS and PEV scores for main forecast experiments are given in Table 1 and discussed in the main text.

The CSS and PEV scores for additional, sensitivity forecast experiments are given in Supplementary Table S7. These experiments are carried out for different NAO indices in the ESO period using the ${{\rm{SIA}}}_{{\rm{joint1}}}^{{\rm{full}}}$ index as the predictor. The four NAO indices shown in Supplementary Fig. S1 are used as predictands. As these indices are strongly interdependent, there is only a little sensitivity to the choice among them. However, slightly higher forecast skill scores are obtained for the PC-based index (CSS = 0.73) and the domain-based index (CSS = 0.72) than for the latitude-based index (CSS = 0.68) or the station-based index (CSS = 0.67). These higher scores may reflect a stronger impact of the Arctic sea ice cover on the northern (stronger) than southern (weaker) NAO center of action since the PC-based and domain-based NAO indices are computed from unnormalised SLP anomalies while the latitude-based and station-based NAO indices are defined as a difference between normalised SLP anomalies.

The forecast skill scores are higher for the DJFM mean NAO indices than for the mean NAO indices computed over shorter timespans, as illustrated in Supplementary Table S7 for the domain-based index. The scores are higher for the late winter (January-February-March; JFM) NAO index (CSS = 0.71) than for the early winter (DJF) NAO index (CSS = 0.63). In the case of 2-month and 1-month mean NAO indices, the highest scores are obtained for the January-February (CSS = 0.62) and January (CSS = 0.57) indices, respectively.

The piecewise linear detrending of the sea ice data with the breakpoint in 2004 (“BP2004” experiments in Supplementary Table S7) results in significantly higher forecast skill scores than the simple linear detrending of these data over the full ESO period (“linear” experiments in Supplementary Table S7). For instance, using the “linear” method instead of the “BP2004” method reduces the CSS value of the forecast of the PC-based DJFM NAO index from 0.73 to 0.64. The forecast of the domain-based JFM and DJF NAO indices using the “linear” method yields CSS values of 0.63 and 0.54, respectively.

## Electronic supplementary material

Supplementary figures and tables
