# Supplementary material for: Climate impacts and Arctic precursors of changing storm track activity in the Atlantic-Eurasian region
Source: Sci Rep. 2018 Dec 12;8:17786. doi: 10.1038/s41598-018-35900-8 (PMC6290774; doi:10.1038/s41598-018-35900-8)
Supplement: Supplementary file 1 — Supplementary figures and tables [file 41598_2018_35900_MOESM1_ESM.pdf]

Climate impacts and Arctic precursors of changing storm track activity in the Atlantic-Eurasian region

Pawel Schlichtholz
Institute of Oceanology
Polish Academy of Sciences
Powstancow Warszawy 55
81-712 Sopot, Poland
e-mail: schlicht@iopan.gda.pl

November 19, 2018

This document contains the following supplementary information:

(i) Supplementary Figures S1-S5 (pages 2-6)

(ii) Supplementary Tables S1-S7 (pages 7-13)

(i) Supplementary Figures

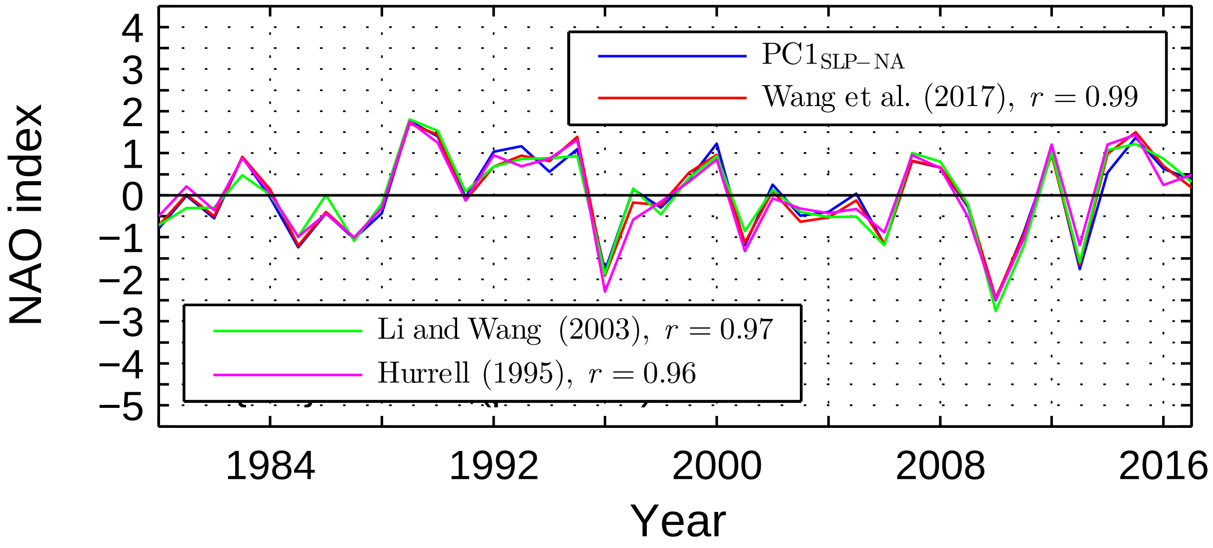

Figure S1: **Indices of the wintertime (DJFM) North Atlantic Oscillation (NAO) during the ESO period.** The selected indices are (blue curve) the first principal component time series $PC1_{SLP-NA}$ of the sea level pressure (SLP) variability in the North Atlantic region (20°-80°N, 90°W-40°E), (red curve) domain-based NAO index defined as the difference between the SLP anomalies averaged over [25°-50°N, 50°W-10°E] and [55°-85°N, 40°W-20°E] boxes, (green curve) latitude-based NAO index defined as the difference of normalised SLP between two latitude zones (35° and 65°N) averaged over the longitudes of 80°W-30°E, and (magenta curve) station-based NAO index representing the difference of normalised SLP between Lisbon, Portugal and Stykkisholmur/Reykjavik. All indices are based on linearly detrended data. Years correspond to the January. Correlation r is the correlation of the given index with $PC1_{SLP-NA}$.

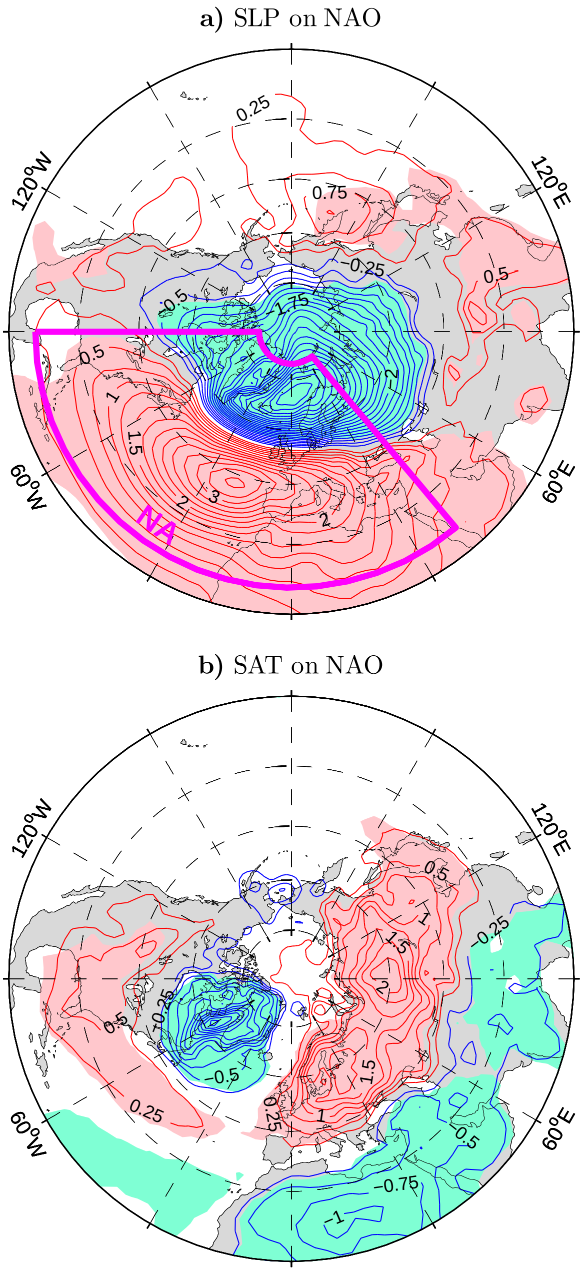

Figure S2: **Surface climate variability associated with the wintertime (DJFM) North Atlantic Oscillation (NAO) during the ESO period (1980-2017, years of the January).** **a** and **b**, Detrended anomalies of the sea level pressure (SLP) and surface air temperature (SAT), respectively, regressed onto the NAO index defined as the first principal component time series of SLP variability in the North Atlantic region (blue curve in Fig. 1c). Red and blue contours represent positive and negative anomalies, respectively. The CI is 0.25 hPa and 0.25 K per unit NAO index, respectively. Pink and aquamarine shading denote, respectively, positive and negative anomalies statistically significant at the 95% confidence level. The North Atlantic region is marked by a magenta box in **a**. The maps were generated by MathWorks MATLAB R2014a with M_Map (http://www.eoas.ubc.ca/~rich/map.html).

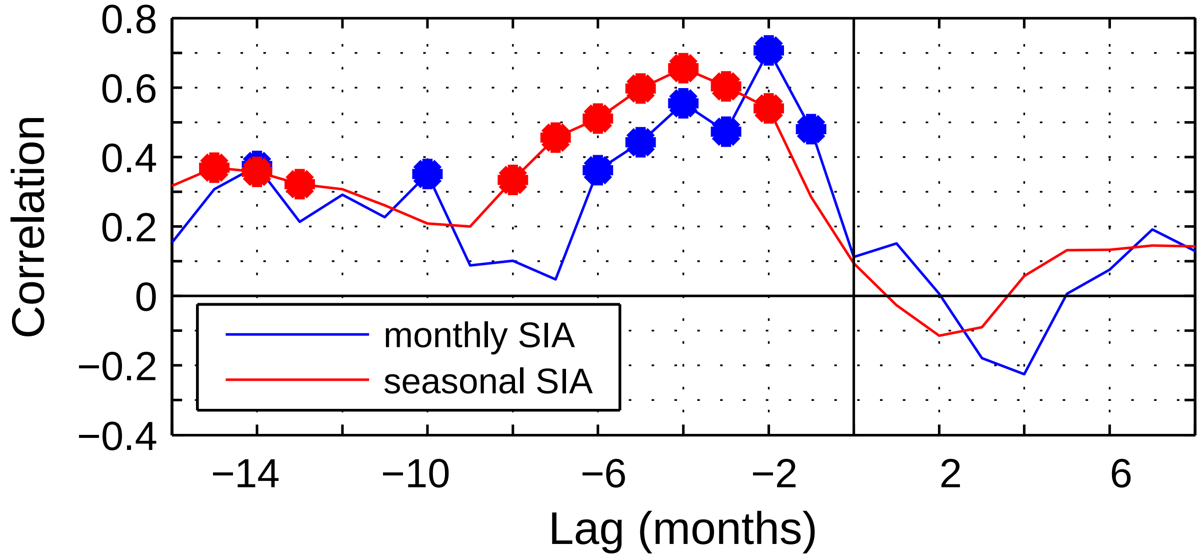

Figure S3: **Time-lagged correlation coefficient of the wintertime (DJFM) STA_{EA} index with the sea ice area (SIA) anomalies in the northern Barents/Kara Sea region during the ESO period.** The filled circles denote correlations statistically significant at the 95% confidence level. Negative lags correspond to SIA anomalies leading the STA_{EA} index. Lag -2 months for the blue curve corresponds to October. Lag -4 months for the red curve corresponds to early autumn (ASON). The STA_{EA} index is the PC1 of storm track activity variations over Eurasia (red curve in Fig. 1c). The SIA was obtained by integration of sea ice concentration over the NBKS box in Fig. 7a. The SIA time series were piecewise linearly detrended with the break point in 2004.

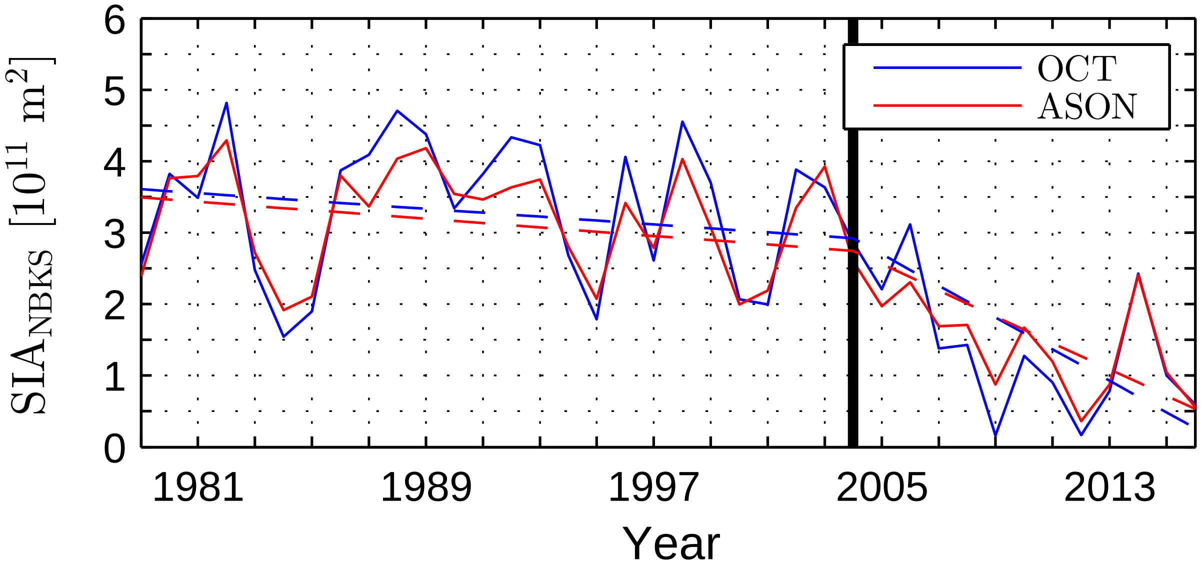

Figure S4: **Time series of the sea ice area (SIA) in the northern Barents/Kara Sea region during the ESO period.** Solid blue and red curves show the mean SIA in October and early autumn (ASON), respectively. Dashed lines show the corresponding continuous piecewise linear trends with the breakpoint in 2004. The SIA was obtained by integration of sea ice concentration over the NBKS box in Fig. 7a.

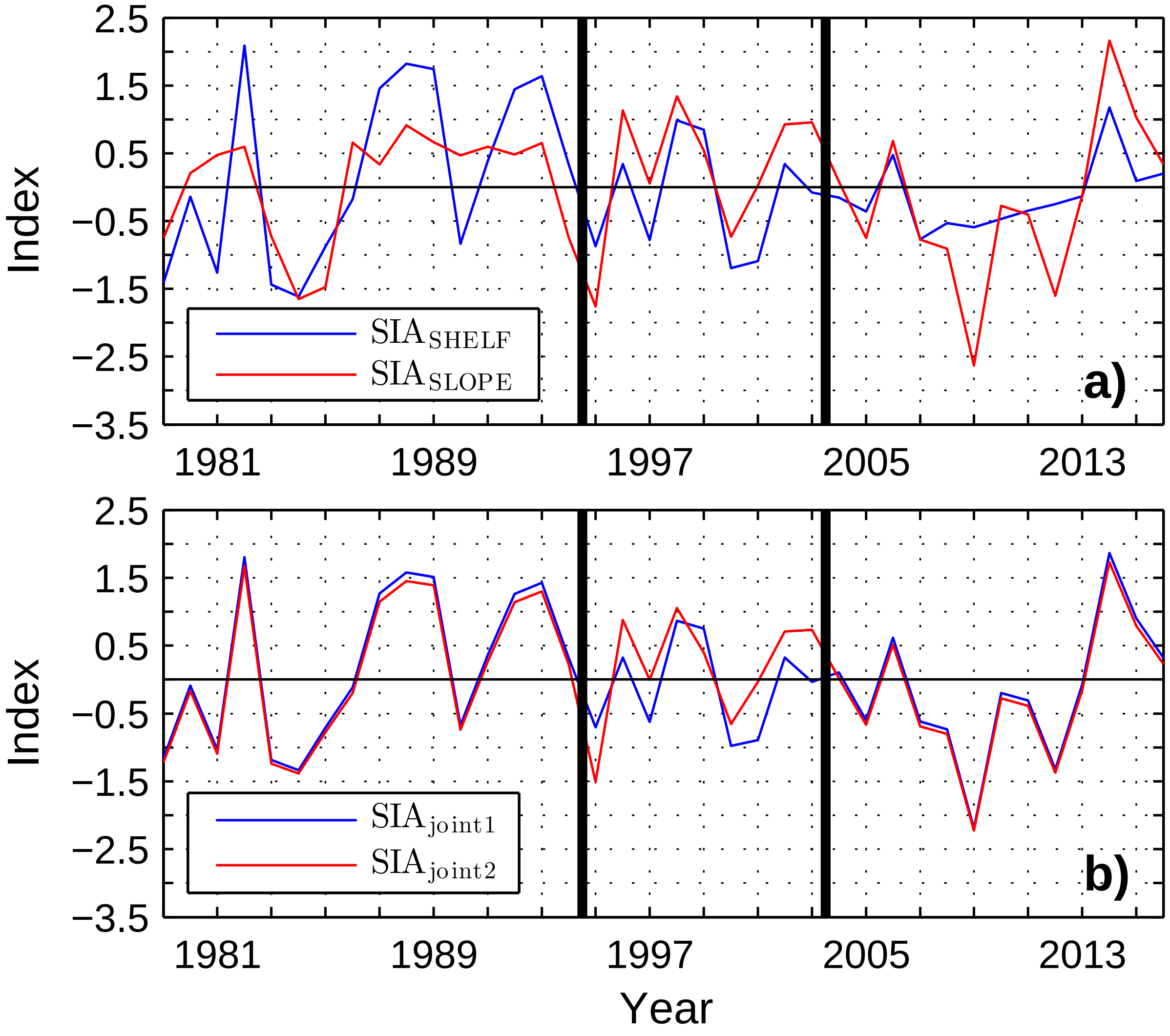

Figure S5: **Indices of sea ice cover variability in the northern Barents/Kara Sea region in October during the ESO period.** **a**, The SIA_{SHELF} (blue curve) and SIA_{SLOPE} (red curve) indices obtained by integration of the sea ice concentration over the SHELF box in Fig. 9a and SLOPE box in Fig. 9b, respectively, followed by subtraction of a continuous piecewise linear trend with the breakpoint in 2004 from the time series. **b**, The SIA_{joint1} (blue curve) and SIA_{joint2} (red curve) indices obtained by merging of the SIA_{SHELF} and SIA_{SLOPE} indices followed by a renormalization of the time series. The SIA_{joint1} (resp. SIA_{joint2}) index is based on the anomalies of SIA_{SHELF} from the period 1979-2003 (resp. 1979-1994) and the anomalies of SIA_{SLOPE} afterwards. The black vertical lines indicate the merging points.

(ii) Supplementary Tables

Table S1: **Main acronyms, abbreviations and indices.**

Acronym	Explanation
ASON	early autumn (August-to-November)
CSS	correlation skill score
DJFM	winter (December-to-March)
EOF	empirical orthogonal function
ESO	era of satellite observations; here the 1979-2017 period
GPH	geopotential height
$\mathrm{GPH_{LB}}$	GPH at 300 hPa (Z_{300}) averaged over the Lake Baikal area (LB box in Fig. 4b)
NAO	North Atlantic Oscillation
NH	Northern Hemisphere
OCT	October
PC	principal component
PC1	PC of the first EOF mode
$\mathrm{PC1_{SAT-EA}}$	PC1 of SAT variability in Eurasia (land within the EA box in Fig. 5b)
$\mathrm{PC1_{SLP-NA}}$	PC1 of SLP variability in the North Atlantic region (NA box in Fig. 1a)
$\mathrm{PC1_{STA-EA}}$	PC1 of STA variability over Eurasia (EA box in Fig. 1b)
$\mathrm{PC1_{STA-NA}}$	PC1 of STA variability in the North Atlantic region (NA box in Fig. 1a)
PEV	proportion of explained variance
SIC	sea ice concentration
SIA	sea ice area (integrated SIC)
$\mathrm{SIA_{NBKS}}$	SIA in the northern Barents/Kara Sea region (NBKS box in Fig. 7a)
$\mathrm{SIA_{SHELF}}$	SIA in the northeastern Barents Sea shelf region (SHELF box in Fig. 9a)
$\mathrm{SIA_{SLOPE}}$	SIA in the northern Kara Sea shelf slope region (SLOPE box in Fig. 9b)
$\mathrm{SIA_{joint1}}$	time series of $\mathrm{SIA_{SHELF}}$ and $\mathrm{SIA_{SLOPE}}$ anomalies merged in 2003/2004
$\mathrm{SIA_{joint2}}$	time series of $\mathrm{SIA_{SHELF}}$ and $\mathrm{SIA_{SLOPE}}$ anomalies merged in 1994/1995
SAT	surface air temperature
$\mathrm{SAT_{A}}$	SAT averaged over northern Asia (A box in Fig. 5a)
$\mathrm{SAT_{EA}}$ index	$\equiv \mathrm{PC1_{SAT-EA}}$
SLP	sea level pressure
STA	storm track activity ($\overline{v'v'}_{300}$)
$\mathrm{STA_{EA}}$ index	$\equiv \mathrm{PC1_{STA-EA}}$
$\mathrm{STA_{NE}}$	STA averaged over northern Europe (NE box in Fig. 7b)

Table S2: **Summary information on leading EOF modes of the variability in wintertime (DJFM) storm track activity (STA; $\overline{v'v'}_{300}$) and sea level pressure (SLP) over the North Atlantic region during the ESO period (1980-2017, years of the January).** All EOFs are computed for the same area (NA box in Fig. 1a) using raw (nondetrended) data. EOFn denotes the EOF of order n. Columns VAR and s give the variance (in %) explained by the given mode and the corresponding significance parameter, respectively. If $s < 1$ (resp. $s > 1$), the mode is independent from (resp. contaminated by) other modes (see Methods). Column r(NAO) gives the correlation of the PC time series of the given EOF with the concurrent NAO index (PC of the first SLP mode). The correlations are given only if significant at the 95% confidence level. Correlations significant at the 99.9% confidence level are in boldface and italic.

Field	EOFn	VAR	s	r(NAO)
STA	EOF1	24.6	3.13	***0.74***
STA	EOF2	21.2	3.13	-
SLP	EOF1	52.2	0.44	***1***
SLP	EOF2	16.2	1.24	-

Table S3: **Summary information on first leading EOF modes of the variability of selected wintertime (DJFM) atmospheric fields in selected areas of the Atlantic-Eurasian region during the ESO period (1980-2017, years of the January).** The atmospheric fields are the storm track activity (STA; $\overline{v'v'}_{300}$), sea level pressure (SLP) and surface air temperature (SAT). The STA mode is computed for the North Atlantic region (NA box in Fig. 1a) and for Eurasia poleward of 30°N (EA box in Fig. 1b). The SLP mode is computed for the North Atlantic region. The SAT mode is computed for Eurasia poleward of 30°N (land within the EA box in Fig. 5b). Prior to the EOF decomposition, all fields were linearly detrended. Columns VAR and s give the variance (in %) explained by the first mode in the given area and the corresponding significance parameter, respectively. If $s < 1$, the mode is independent from the corresponding second mode (see Methods). Column PC1 indicates the notation used for the principal component time series of the selected mode. Columns r(NAO), $r(\mathrm{STA_{EA}})$ and $r(\mathrm{SAT_{EA}})$ give the correlation of the selected PC1 with $\mathrm{PC1_{SLP-NA}}$ (NAO index), $\mathrm{PC1_{STA-EA}}$ ($\mathrm{STA_{EA}}$ index), and $\mathrm{PC1_{SAT-EA}}$ ($\mathrm{SAT_{EA}}$ index), respectively. All correlations are significant at the 99.9% confidence level.

Field	Area	VAR	s	PC1	r(NAO)	$r(\mathrm{STA_{EA}})$	$r(\mathrm{SAT_{EA}})$
STA	NA	25.3	2.14	$\mathrm{PC1_{STA-NA}}$	***0.75***	***0.75***	***0.65***
SLP	NA	52.4	0.44	$\mathrm{PC1_{SLP-NA}}$	***1***	***0.83***	***0.86***
STA	EA	28.2	0.88	$\mathrm{PC1_{STA-EA}}$	***0.83***	***1***	***0.82***
SAT	EA	48.2	0.43	$\mathrm{PC1_{SAT-EA}}$	***0.86***	***0.82***	***1***

Table S4: **Correlation coefficient (×100) of PC-based indices with area-averaged indices of wintertime (DJFM) atmospheric variability during the ESO period (1980-2017, years of the January).** The PC-based indices (listed in the 1st column) are the STA_{EA} index defined as the PC1 of storm track activity variations over Eurasia poleward of 30°N (within the EA box in Fig. 1b), the NAO index defined as the PC1 of sea level pressure variations in the North Atlantic region (within the NA box in Fig. 1a), and the SAT_{EA} index defined as the PC1 of surface air temperature variations in Eurasia poleward of 30°N (over land within the EA box in Fig. 5b). The area-averaged indices (listed in the 1st row) are the STA_{NE} index defined as the storm track activity anomalies averaged over northern Europe (NE box in Fig. 7b), the GPH_{LB} index defined as the geopotential height anomalies averaged over the Lake Baikal area (LB box in Fig. 4b), and the SAT_A index defined as the surface air temperature anomalies averaged over northern Asia (A box in Fig. 5a). All indices are based on linearly detrended data. All correlations are significant at the 99.9% confidence level.

Index	STA_{NE}	GPH_{LB}	SAT_A
$PC1_{STA-EA} \equiv STA_{EA}$	***90***	***76***	***79***
$PC1_{SLP-NA} \equiv$ NAO	***88***	***71***	***74***
$PC1_{SAT-EA} \equiv SAT_{EA}$	***82***	***84***	***95***

Table S5: **Lagged correlation coefficient (×100) of selected PC-based and area-averaged indicators of wintertime (DJFM) atmospheric variability with the previous October indices of sea ice area (SIA) in the Barents/Kara Sea region during the ESO period**. The atmospheric variables (same as in Supplementary Table S4) include the STA_{EA} index ($PC1_{STA-EA}$, red curve in Fig. 1c), STA_{NE} index (STA anomalies averaged over the blue box in Fig. 7b), NAO index ($PC1_{SLP-NA}$, blue curve in Fig. 1c), GPH_{LB} index (GPH anomalies averaged over the black box in Fig. 4b), SAT_{EA} index ($PC1_{SAT-EA}$, blue curve in Fig. 5c) and SAT_{A} index (SAT anomalies averaged over the black box in Fig. 5a). The SIA indices were obtained by integration of sea ice concentration over the NBKS box in Fig. 7a, SHELF box in Fig. 9a and SLOPE box in Fig. 9b. Prior to the correlation analysis, a continuous piecewise linear trend (with the breakpoint in 2004) was removed from the SIA indices. The atmospheric time series were linearly detrended over the full ESO period. Correlations are given for the entire ESO period (1979-2016, years of the October), early ESO period (1979-2003) and late ESO period (2004-2016). All correlations are significant at the 95% confidence level and those significant at the 99% (99.9%) level are in boldface (boldface and italic).

Index	SIA_{NBKS}^{79-16}	SIA_{NBKS}^{79-03}	SIA_{NBKS}^{04-16}	SIA_{SHELF}^{79-16}	SIA_{SLOPE}^{79-16}	SIA_{SHELF}^{79-03}	SIA_{SLOPE}^{04-16}
STA_{EA}	***70***	***65***	**80**	***67***	***68***	***69***	***86***
STA_{NE}	***75***	***72***	***88***	***67***	***75***	***73***	***90***
NAO	***66***	**62**	**73**	***62***	***66***	***71***	***81***
GPH_{LB}	***57***	48	**77**	**48**	***62***	**49**	**82**
SAT_{EA}	***57***	46	**75**	**51**	***61***	**53**	**81**
SAT_{A}	***61***	**49**	**77**	**49**	***66***	48	**78**

Table S6: **Results from Monte Carlo testing of a null hypothesis that there was no strengthening of the link between wintertime (DJFM) atmospheric variations and sea ice anomalies in the preceding October during the late ESO period.** P_{SLOPE} is the percentage of Monte Carlo trials in which the correlation of a given wintertime atmospheric index ($\mathrm{STA_{EA}}$, $\mathrm{STA_{NE}}$, NAO, $\mathrm{GPH_{LB}}$, $\mathrm{SAT_{EA}}$ or $\mathrm{SAT_{A}}$) in the early ESO period (1980-2004, years of the January) with a trial SIA index computed using shuffled $\mathrm{SIA_{SLOPE}}$ anomalies of the preceding October (see Methods) is at least as large as the correlation between the atmospheric index and $\mathrm{SIA_{SLOPE}}$ anomalies in the late ESO period (2004-2016, years of the October $\mathrm{SIA_{SLOPE}}$ index). P_{SHELF} and P_{NBKS} are the corresponding percentages for shuffled $\mathrm{SIA_{SHELF}}$ and $\mathrm{SIA_{NBKS}}$ anomalies as trial SIA indices. For each atmospheric variable, the maximum of P_{SLOPE}, P_{SHELF} and P_{NBKS} is in boldface. The $\mathrm{SIA_{NBKS}}$, $\mathrm{SIA_{SHELF}}$ and $\mathrm{SIA_{SLOPE}}$ indices are defined as piecewise linearly detrended time series (breakpoint in 2004) of the sea ice concentration integrated over the NBKS box in Fig. 7a, SHELF box in Fig. 9a and SLOPE box in Fig. 9b, respectively. The atmospheric indices (same as in Supplementary Tables S4 and S5) are based on data linearly detrended over the full ESO period.

	$\mathrm{STA_{EA}}$	$\mathrm{STA_{NE}}$	NAO	$\mathrm{GPH_{LB}}$	$\mathrm{SAT_{EA}}$	$\mathrm{SAT_{A}}$
P_{SLOPE}	0.5%	1.7%	1.3%	0.7%	0.4%	2.4%
P_{SHELF}	**4.6%**	0.6%	**19.6%**	1.6%	**4.9%**	9.5%
P_{NBKS}	1.8%	**2.2%**	4.0%	**1.8%**	2.5%	**10.0%**

Table S7: **Correlation skill score (CSS) and the proportion of explained variance (PEV) in sensitivity experiments with the leave-1-yr-out cross-validation forecast of the wintertime (DJFM) NAO index in the ESO period from the $\mathbf{SIA_{joint1}}$ index of the preceding October.** The SIA_{joint1} index is obtained by merging the time series of SIA_{SHELF} anomalies from the early ESO period (1979-2003) with the SIA_{SLOPE} anomalies from the late ESO period (2004-2016). The SIA_{SHELF} and SIA_{SLOPE} time series are obtained by integration of the sea ice concentration over the SHELF box in Fig. 9a and SLOPE box in Fig. 9b, respectively. To construct the SIA_{joint1} index either a continuous piecewise linear trend with the breakpoint in 2004 (d_{SIA} = "BP2004") or the linear trend over the full ESO period (d_{SIA} = "linear") was removed from the time series of SIA_{SHELF} and SIA_{SLOPE}. Results are given for different (PC-based, domain-based, latitude-based and station-based) NAO indices (all linearly detrended; see Methods for their definition) and different parts of the winter season. Results from the forecasts of the 2-month and 1-month mean domain-based NAO indices are given only for the months (JF and J) with the highest skill scores obtained. All CSS values are significant at the 99.9% confidence level.

Index	Season	d_{SIA}	CSS	PEV
PC-based	DJFM	BP2004	***0.73***	0.53
Domain-based	DJFM	BP2004	***0.72***	0.51
Latitude-based	DJFM	BP2004	***0.68***	0.46
Station-based	DJFM	BP2004	***0.67***	0.44
Domain-based	DJF	BP2004	***0.63***	0.40
Domain-based	JFM	BP2004	***0.71***	0.50
Domain-based	JF	BP2004	***0.62***	0.38
Domain-based	J	BP2004	***0.57***	0.32
PC-based	DJFM	linear	***0.64***	0.40
Domain-based	DJFM	linear	***0.61***	0.37
Domain-based	DJF	linear	***0.54***	0.29
Domain-based	JFM	linear	***0.63***	0.39
